# Pentachlorophenol enhances bladder cancer cell invasion through altered protein stability

**DOI:** 10.1016/j.isci.2026.117357

**Published:** 2026-08-26

**Authors:** Ranran Zhou, Wansong Zhang, Ming Xia, Ker Zhang, Wei Luo, Tianhang Zhu, Cheng Yang, Cundong Liu

**Affiliations:** 1 Department of Urology, The Third Affiliated Hospital, Southern Medical University, Guangzhou, Guangdong Province 510630, China; 2 The Third Clinical School of Southern Medical University, Guangzhou, Guangdong Province 510630, China; 3 Department of Urology, The Seventh Affiliated Hospital, Southern Medical University, Foshan, Guangdong Province 528244, China

**Keywords:** bladder cancer, pentachlorophenol, EGFR, MAP2K1, NFE2L2

## Abstract

Pentachlorophenol (PCP) is a persistent environmental contaminant, but its effect on bladder cancer invasiveness is unclear. Here, we show that environmentally relevant PCP concentrations enhance invasion of T24 and SW780 bladder cancer cells, with limited effects on migration or proliferation. Integrative target prediction, transcriptomics, and machine learning identified EGFR, MAP2K1, and NFE2L2 as candidate contributors. PCP altered the protein stability of these factors, and EGFR or MAP2K1 knockdown or NFE2L2 overexpression partially attenuated PCP-induced invasion. A three-gene signature based on these factors was associated with poor prognosis across five bladder cancer cohorts, although these datasets lacked PCP exposure information. Molecular docking and molecular dynamics simulations predicted compatible interactions between PCP and the three proteins. Together, these findings identify candidate molecular contributors to PCP-associated bladder cancer invasion and support further chronic-exposure and *in vivo* studies.

## Highlights

• Environmentally relevant PCP levels promote BLCA cell invasion
• EGFR, MAP2K1, and NFE2L2 are linked to poor prognosis in BLCA
• PCP alters EGFR, MAP2K1, and NFE2L2 protein stability

## Graphical abstract

**Figure:**
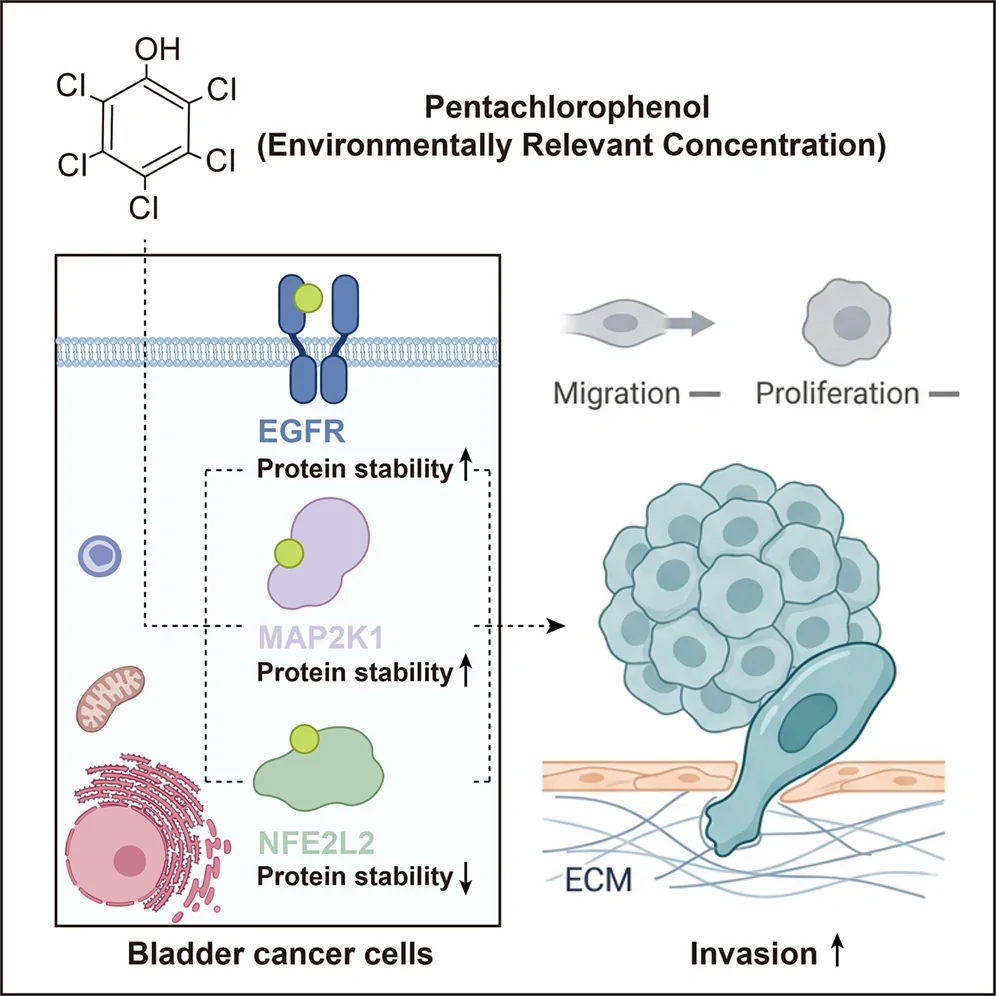

## Teaser

Cancer; Molecular interaction; Therapeutics

## Introduction

Bladder cancer (BLCA) is a leading global urinary tract malignancy, with approximately 630,000 new cases and 220,000 deaths annually. Its etiology involves both genetic susceptibility and environmental factors, with established risks including tobacco smoke and occupational exposure to aromatic amines or polycyclic aromatic hydrocarbons.^1^^,^^2^ Epidemiological studies further associate BLCA with other environmental contaminants, such as disinfection by-products, arsenic, and airborne pollutants like benzene and toluene.^3^^,^^4^^,^^5^ While such exposures highlight the importance of toxicant reduction for prevention, the role of other pervasive environmental carcinogens in BLCA progression remains inadequately explored. Pentachlorophenol (PCP), a persistent organic pollutant with known carcinogenic potential in other tissues, represents a compelling candidate for such investigation.

PCP, a chlorinated organic compound previously widely used as a wood preservative and biocide, persists in the environment due to its stable carbon-chlorine bonds. It bioaccumulates in humans, with detectable levels found in blood, urine, and other tissues across diverse populations.^6^^,^^7^ Exposure varies considerably; for instance, individuals in log homes preserved with PCP show serum concentrations up to 5 μM, compared with ∼0.15 μM in the general population.^8^^,^^9^ A Chinese cohort study detected PCP in over 90% of serum samples, with levels reaching approximately 1.14 μM.^10^ Such pervasive exposure is concerning, given associations between PCP and adverse health outcomes, including thyroid dysfunction, neurodevelopmental effects, and cancers such as non-Hodgkin lymphoma.^11^^,^^12^^,^^13^ One proposed oncogenic mechanism involves mitogen-activated protein kinase (MAPK) pathway-mediated upregulation of pro-inflammatory cytokines such as interleukin 1 beta (IL-1β) and interleukin 6 (IL-6), which may foster a tumor-promoting microenvironment.^8^ While PCP is recognized as a potential carcinogen, its specific role and molecular mechanisms in BLCA progression remain unclear. Importantly, whether environmentally relevant concentrations of PCP can directly influence malignant phenotypes in BLCA cells has not been established, highlighting the need for mechanistic studies to clarify its impact on BLCA pathogenesis.

Therefore, this study was designed to test the hypothesis that environmentally relevant concentrations of PCP promote the malignancy of BLCA cells through specific dysregulation of key signaling pathways. We assessed key malignant phenotypes, invasion, migration, and proliferation, in BLCA cell lines (T24 and SW780) following exposure to 0.05, 0.5, or 5 μM PCP. Putative targets of PCP were identified through an integrative bioinformatics analysis of toxicological and cancer databases and further refined using a machine learning-based prognostic framework. Molecular docking (MD) and molecular dynamics simulations (MDS) were employed to evaluate candidate interactions, which were subsequently validated by measuring mRNA and protein stability of key targets after PCP treatment. Functional assays using transfection approaches were conducted to confirm the mechanistic involvement of identified targets. Our findings reveal a novel mechanistic link between PCP exposure and BLCA progression.

## Results

### PCP promoting BLCA cell invasion without affecting migration or proliferation

**Figure 1:**
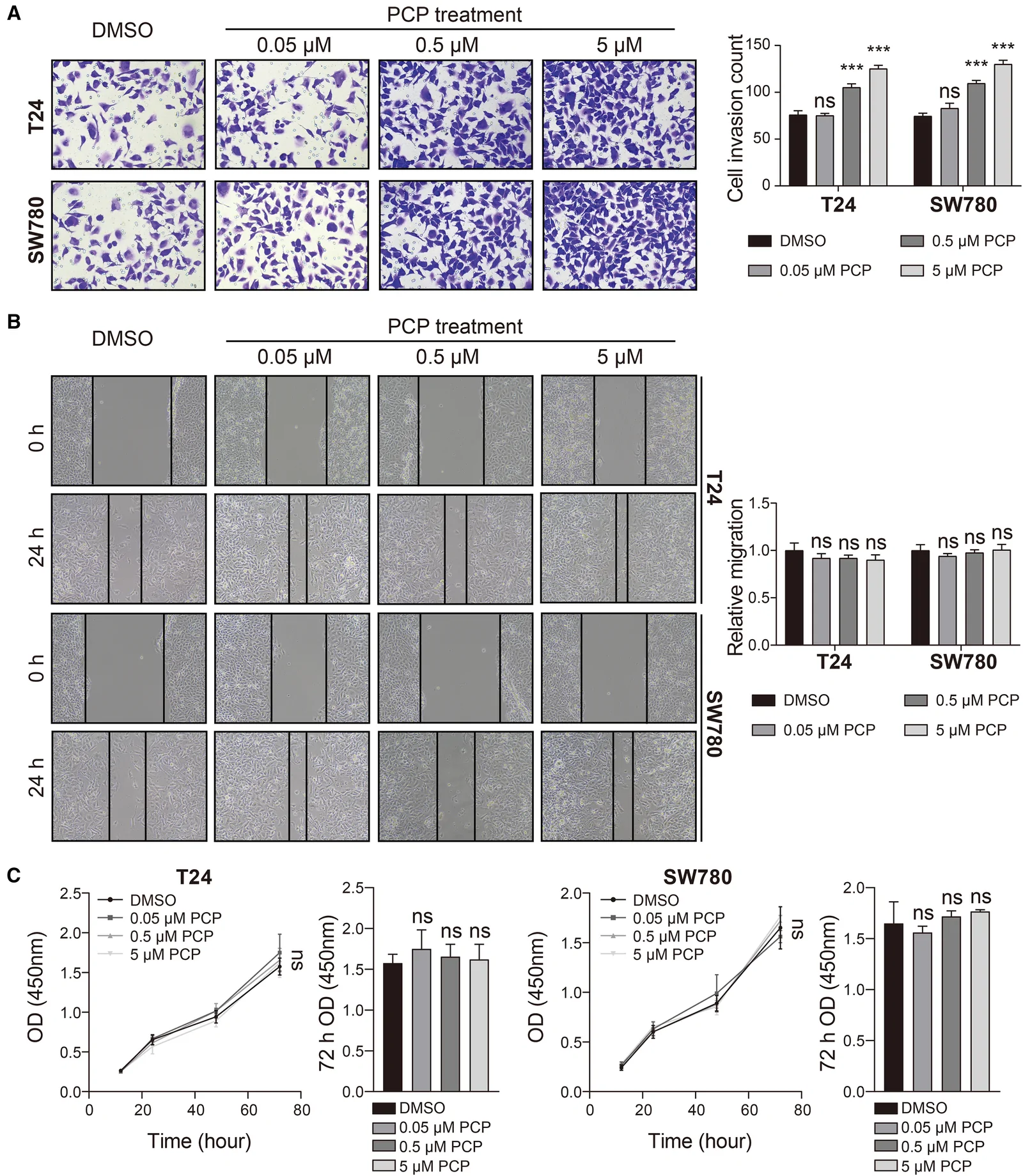
Environmentally relevant levels of PCP selectively promote BLCA cell invasion The effect of 0.05, 0.5, and 5 μM PCP on the invasion (A), migration (B), and proliferation (C) of T24 and SW780 cells. Values are presented as mean ± SD from at least three independent experiments (*n* = 3 biological replicates). ns, not significant; ∗∗∗*p* < 0.001. PCP, pentachlorophenol.

While previous epidemiological studies have linked PCP exposure to the risk of certain cancers,^14^^,^^15^^,^^16^ its role in BLCA remains unclear. Thus, in the initial phase of this study, we assessed the influence of environmentally relevant concentrations of PCP (0.05, 0.5, and 5 μM)^8^^,^^9^^,^^10^ on invasion, migration, and proliferation in BLCA cells. A 24-h exposure to 0.05 μM PCP showed no effect on T24 and SW780 cells (all *p* > 0.05, Figures 1A–1C). However, at 0.5 and 5 μM, PCP significantly increased cellular invasion in a dose-dependent manner (all *p* < 0.001, Figure 1A), without altering migration or proliferation (all *p* > 0.05, Figures 1B and 1C). These findings reveal that PCP can specifically enhance the invasiveness of BLCA cells at environmentally relevant exposure levels, independent of migratory or proliferative changes.

To evaluate the specificity of PCP-induced invasiveness, we tested a structurally related chlorophenol, 2,4-dichlorophenol (2,4-DCP), which shares the chlorinated phenol backbone but exhibits lower carcinogenic potential than PCP. Treatment of T24 and SW780 cells with 0.05, 0.5, or 5 μM 2,4-DCP for 24 h did not significantly alter cellular invasion capacity relative to vehicle control across all tested concentrations (all *p* > 0.05, Figure S1). These findings suggest that the pro-invasive effect observed with PCP is not attributable to generalized chlorophenol toxicity or non-specific oxidative stress responses but rather reflects a PCP-specific biological activity.

### Detecting the targets of PCP

**Figure 2:**
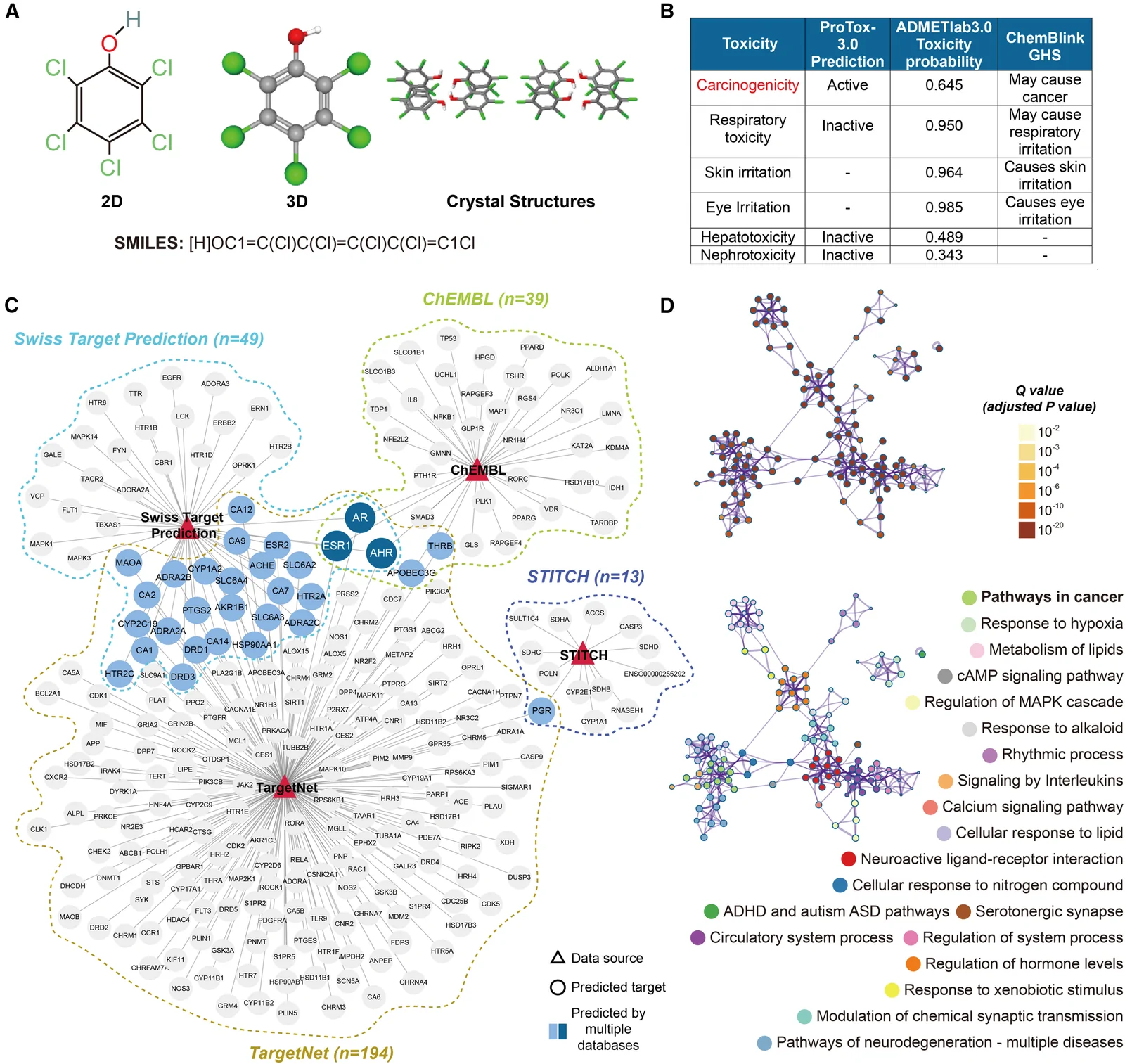
The potential targets of PCP (A) 2D, 3D, and crystal structures of PCP, which were obtained from the PubChem. (B) *In silico* carcinogenicity predictions of PCP generated using ADMETlab 3.0, ProTox 3.0, and ChemBlink. (C) Potential target genes of PCP identified via SwissTargetPrediction, ChEMBL, TargetNet, and STITCH. (D) Functional enrichment analysis of the combined predicted targets of PCP performed with Metascape. 2D, two-dimensional; 3D, three-dimensional.

To identify potential targets of PCP, we performed a multi-database analysis. We obtained the structure of PCP from PubChem (Figure 2A). Toxicity profiling using ADMETlab 3.0, ProTox 3.0, and ChemBlink consistently indicated carcinogenic properties (Figure 2B), aligning with epidemiological data on PCP exposure and cancer risk. For target prediction, TargetNet, SwissTargetPrediction, STITCH, and ChEMBL were employed, yielding 194, 49, 13, and 39 targets, respectively (Figure 2C and Table S1). After removing 33 duplicates, 262 unique genes were retained, among which AR, AHR, and ESR1 were commonly identified by SwissTargetPrediction, ChEMBL, and TargetNet (Figure 2C). Functional enrichment indicated that these 262 targets of PCP are enriched in cancer-related pathways such as hypoxia, lipid metabolism, cAMP signaling, and MAPK signaling (Figure 2D).

### Collection of BLCA-related genes

**Figure 3:**
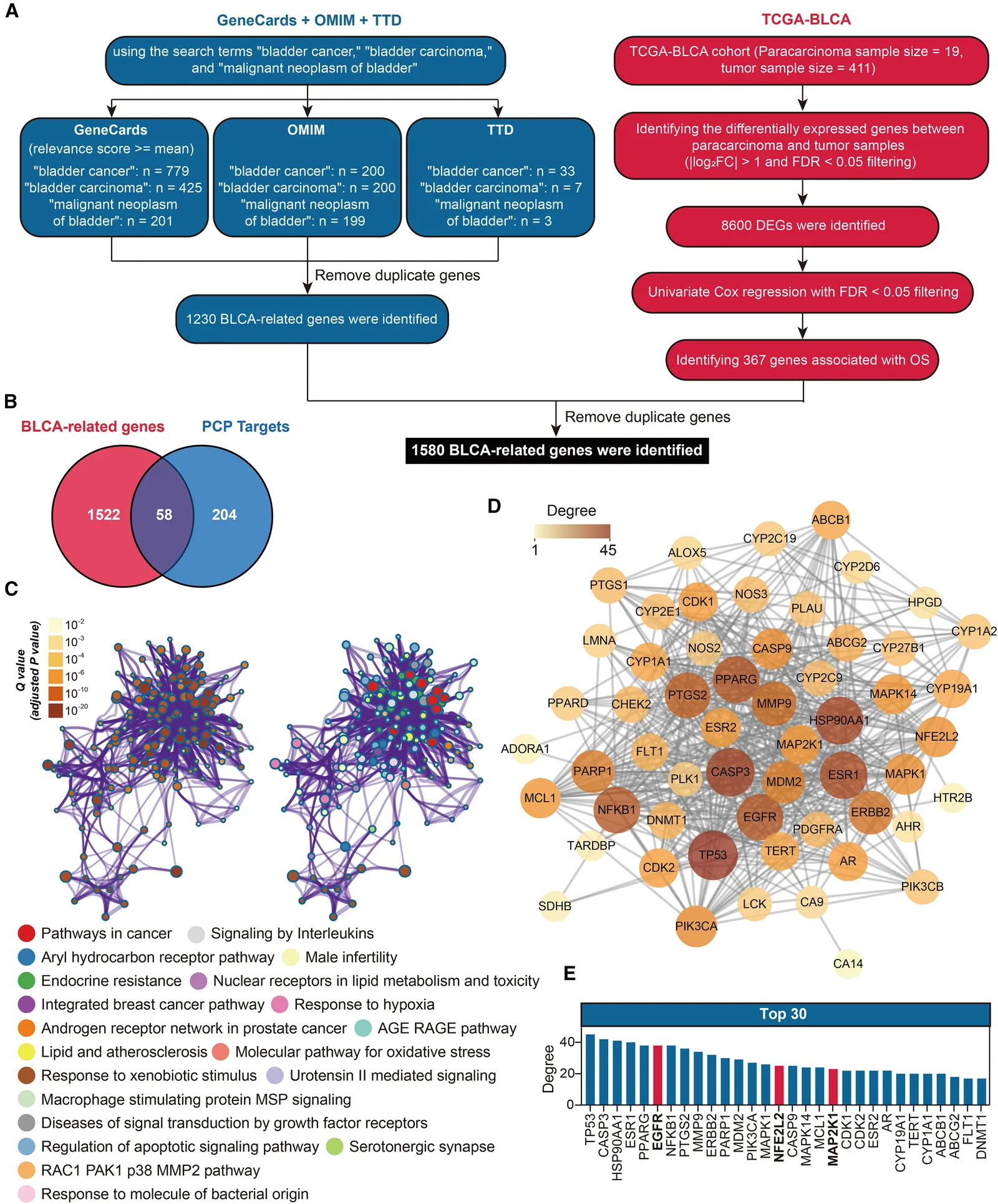
Identification of 58 BLCA-PCP target genes (A) A total of 1,580 BLCA-related genes were obtained from GeneCards, OMIM, TTD, and TCGA. (B) 58 genes were identified as the potential BLCA-PCP target genes. (C) Functional enrichment analysis of the 58 BLCA-PCP target genes performed using Metascape. (D) The PPI network of identified 58 genes. (E) The top 30 genes with the highest connectivity degree in the PPI network. TCGA, the Cancer Genome Atlas; OMIM, online Mendelian inheritance in man; TTD, therapeutic target database; PPI, protein-protein interaction.

We compiled a list of BLCA-related genes from multiple sources. Using the keywords “bladder cancer,” “bladder carcinoma,” and “malignant neoplasm of bladder,”^17^ we retrieved candidate genes from GeneCards, online Mendelian inheritance in man (OMIM), and therapeutic target database (TTD). Genes with a relevance score equal to or above the median were selected from GeneCards.^18^ After removing duplicates, 790, 462, and 33 BLCA-associated genes were obtained from GeneCards, OMIM, and TTD (Table S2), respectively. Additionally, RNA sequencing (RNA-seq) data from the Cancer Genome Atlas (TCGA), comprising 19 normal and 411 tumor BLCA samples, were analyzed. Differential expression analysis identified 8,600 genes, including 5,631 upregulated and 2,969 downregulated genes (Table S3). Univariate Cox regression analysis with false discovery rate (FDR) < 0.05 revealed 367 genes significantly associated with overall survival (OS) (Table S4). Integration of all gene sets after duplicate removal yielded a final list of 1,580 BLCA-related genes (Figure 3A).

### Identification of BLCA-PCP targets

Through intersection analysis of 1,580 BLCA-associated genes and 262 PCP targets, 58 overlapping genes were identified as BLCA-PCP target genes (Figure 3B). Functional enrichment analysis revealed their primary involvement in cancer-related pathways, interleukin signaling, lipid metabolism, and oxidative stress (Figure 3C). A protein-protein interaction (PPI) network demonstrated strong connectivity among these genes (Figure 3D), and the top 30 hub genes were detected (Figure 3E and Table S5).

### EGFR, MAP2K1, and NFE2L2 identified as BLCA-PCP hub targets via machine learning algorithms

**Figure 4:**
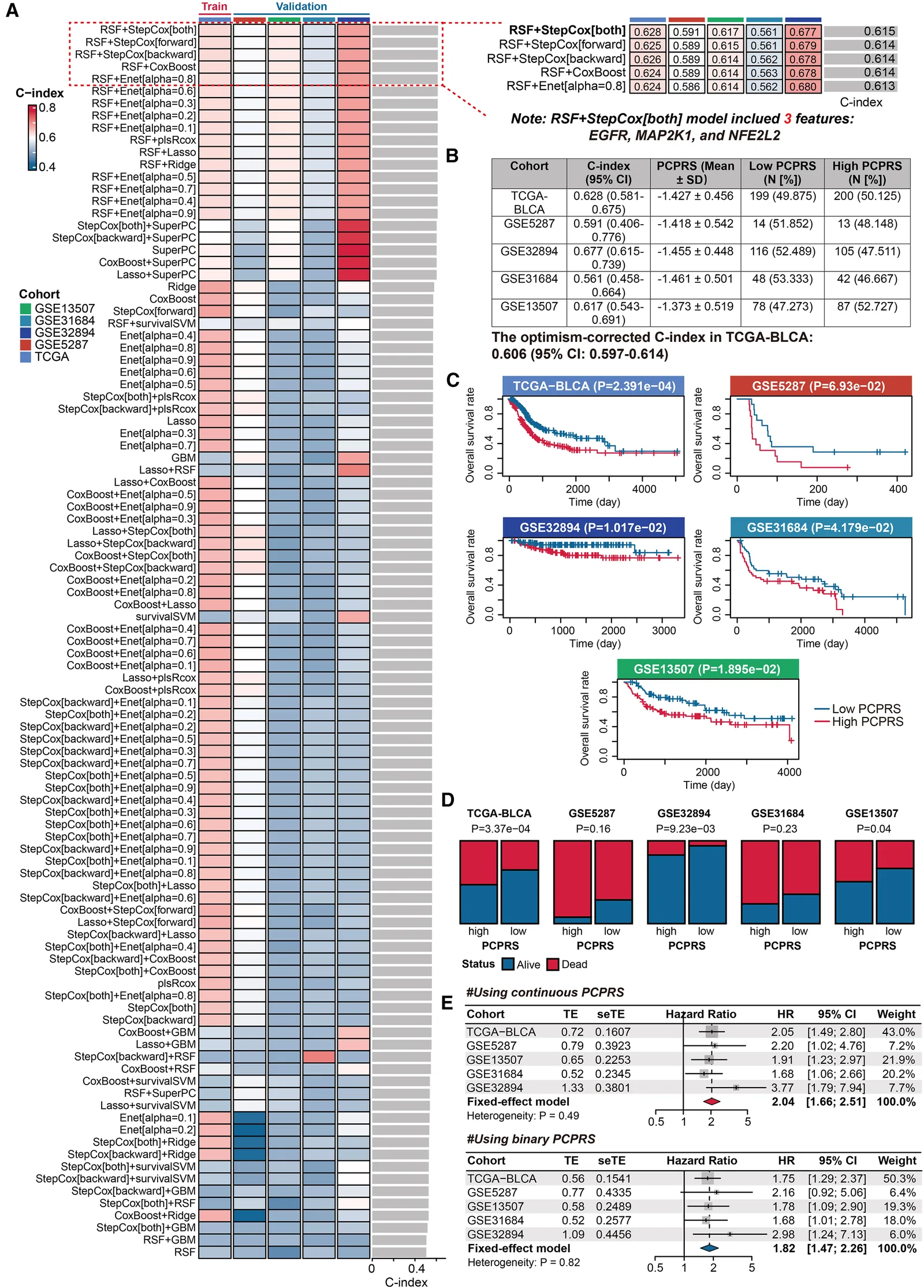
Identification of prognostic genes from 58 BLCA-PCP target genes using 101 machine-learning algorithms (A) Heatmap of the C-index across 101 machine learning algorithms in the training and validation cohorts. The RSF+StepCox[both] model, comprising EGFR, MAP2K1, and NFE2L2, was selected as the optimal prognostic model. (B) Based on the median PCPRS value derived from the training cohort (TCGA-BLCA), patients were stratified into low- and high-PCPRS subgroups. (C) Kaplan-Meier survival indicating the predictive value of PCPRS to OS in the training and validation cohorts. (D) Association between PCPRS stratification and survival statuses in the training and validation cohorts. (E) Meta-analyses conducted to pool the HRs of PCPRS across multiple BLCA cohorts, using the continuous (up) or binary (down) PCPRS for analysis. C-index, concordance index; HRs, hazard ratios; RSF, random survival forest; PCPRS, PCP-related score; EGFR, epidermal growth factor receptor; MAP2K1, mitogen-activated protein kinase kinase 1; NFE2L2, NFE2 like BZIP transcription factor 2; OS, overall survival.

Based on the expression of 58 BLCA-PCP target genes, we evaluated their prognostic relevance in BLCA patients using 101 machine learning algorithms. The TCGA-BLCA cohort served as the training set, while four independent cohorts (GSE13507, GSE31684, GSE32894, and GSE5287) were selected for validation. The baseline clinicopathological features of these cohorts can be found in Table S6. Prior to analysis, batch effect correction was applied to these cohorts (Figure S2). Among all models, Random Survival Forest + Stepwise Cox [both] (RSF + StepCox [both]) achieved the highest average concordance index (C-index) (0.615) and was chosen to calculate the PCP-related score (PCPRS) based on epidermal growth factor receptor (EGFR), mitogen-activated protein kinase kinase 1 (MAP2K1), and nuclear factor erythroid 2-related factor 2 (NFE2L2) (Figure 4A and Table S7). To rigorously validate the model and address potential overfitting, we performed two rounds of internal validation in the TCGA-BLCA training cohort. First, 10-fold cross-validation yielded a C-index of 0.608 (95% confidence interval [CI]: 0.599–0.617), consistent with the apparent performance. Second, 1,000-iteration bootstrap resampling produced an optimism-corrected C-index of 0.606 (95% CI: 0.597–0.614), which closely aligned with the apparent C-index, confirming minimal overfitting and strong internal validity (Figure 4B). We further evaluated the model performance in all external validation cohorts. The C-index with 95% CIs for each cohort was displayed on Figure 4B. The moderate but consistent C-index values across independent cohorts support the generalizability of the PCPRS model.

Using the median PCPRS value (−1.48) from the TCGA-BLCA cohort, patients were stratified into low- and high-PCPRS subgroups. The use of a training-set-derived median cutoff for risk stratification across independent validation cohorts is an established approach in transcriptome-based prognostic modeling,^19^^,^^20^^,^^21^ as it preserves the relative ranking of patients within each cohort and avoids data leakage from re-optimizing cutoffs on validation sets. High PCPRS correlated with adverse clinicopathological characteristics, including advanced tumor grade (*p* < 0.01), Tumor-node-metastasis (TNM) stage (*p* < 0.05), and pathological N (pN) stage (*p* < 0.05, Figure S3A). It also served as an independent predictor of OS in both univariate (hazard ratio [HR] = 1.95; 95% CI: 1.19–3.18; *p* < 0.01) and multivariate (HR = 1.78; 95% CI: 1.07–2.96; *p* < 0.05; Figure S3B) Cox analyses, outperforming conventional clinical variables. Kaplan–Meier analysis confirmed significantly worse OS in the high-PCPRS group across all cohorts except GSE5287 (all *p* < 0.05), where limited sample size (*n* = 27) likely reduced statistical power (*p* > 0.05, Figure 4C). To evaluate whether the null result of the GSE5287 cohort was attributable to limited statistical power, we performed a post-hoc power analysis. Based on the observed Z-statistics of 1.779 and a two-sided alpha level of 0.05, the calculated statistical power was 0.4284, indicating an approximately 43% chance of detecting a true effect. This low power suggests that the non-significant finding in the GSE5287 cohort is likely due to insufficient sample size rather than a true absence of association. PCPRS’s association with survival status was also analyzed in each cohort (Figure 4D). To further evaluate the calibration performance of PCPRS, we generated calibration plots for 1-, 3-, and 5-year OS across all cohorts using the riskRegression R package. For the GSE5287 cohort, which had a maximum follow-up time of 420 days (∼1.2 years), only the 1-year OS calibration plot was generated. The calibration curves demonstrated good agreement between predicted and observed survival probabilities in most cohorts, supporting the reliability of PCPRS for survival prediction (Figure S4). A subsequent meta-analysis incorporating all cohorts confirmed that both continuous (pooled HR = 2.04; 95% CI: 1.66–2.51) and binary (pooled HR = 1.82; 95% CI: 1.47–2.26; Figure 4E) PCPRS were significantly associated with OS. In summary, through extensive machine learning screening, we identified EGFR, MAP2K1, and NFE2L2 as top candidate mediators of PCP-driven BLCA progression among the 58 BLCA-PCP overlapping genes.

### High expressions of EGFR and MAP2K1 and low expression of NFE2L2 associated with unfavorable prognosis in BLCA

We then evaluated the prognostic significance of EGFR, MAP2K1, and NFE2L2 individually in BLCA. Meta-analyses across five independent BLCA cohorts revealed that high expression of EGFR or MAP2K1, or low expression of NFE2L2, was generally associated with poorer OS, with pooled HRs of 1.83 (95% CI: 1.44–2.33) for EGFR, 1.75 (95% CI: 1.36–2.24) for MAP2K1, and 0.59 (95% CI: 0.35–0.98) for NFE2L2 (Figure S5A). At the protein level, the HPA database showed elevated EGFR and MAP2K1 expression and reduced NFE2L2 expression in BLCA tissues in comparison with normal samples (Figure S5B). The association of PCPRS, EGFR, MAP2K1, and NFE2L2 with 24 pan-cancer invasion-related genes^22^^,^^23^ is displayed in Figure S5C. To explore biological functions, enrichment analysis of the top 100 correlated genes from GEPIA (Table S8) indicated that EGFR was linked to cell differentiation and epigenetic regulation, MAP2K1 to cell cycle and translational control, and NFE2L2 to mitophagy, proteostasis, and cell cycle regulation (Figure S6). These analyses indicated that EGFR, MAP2K1, and NFE2L2 may mediate the effects of environmentally relevant PCP concentrations on BLCA invasiveness.

### MD and MDS supporting stable binding of PCP to EGFR, MAP2K1, and NFE2L2 proteins

**Figure 5:**
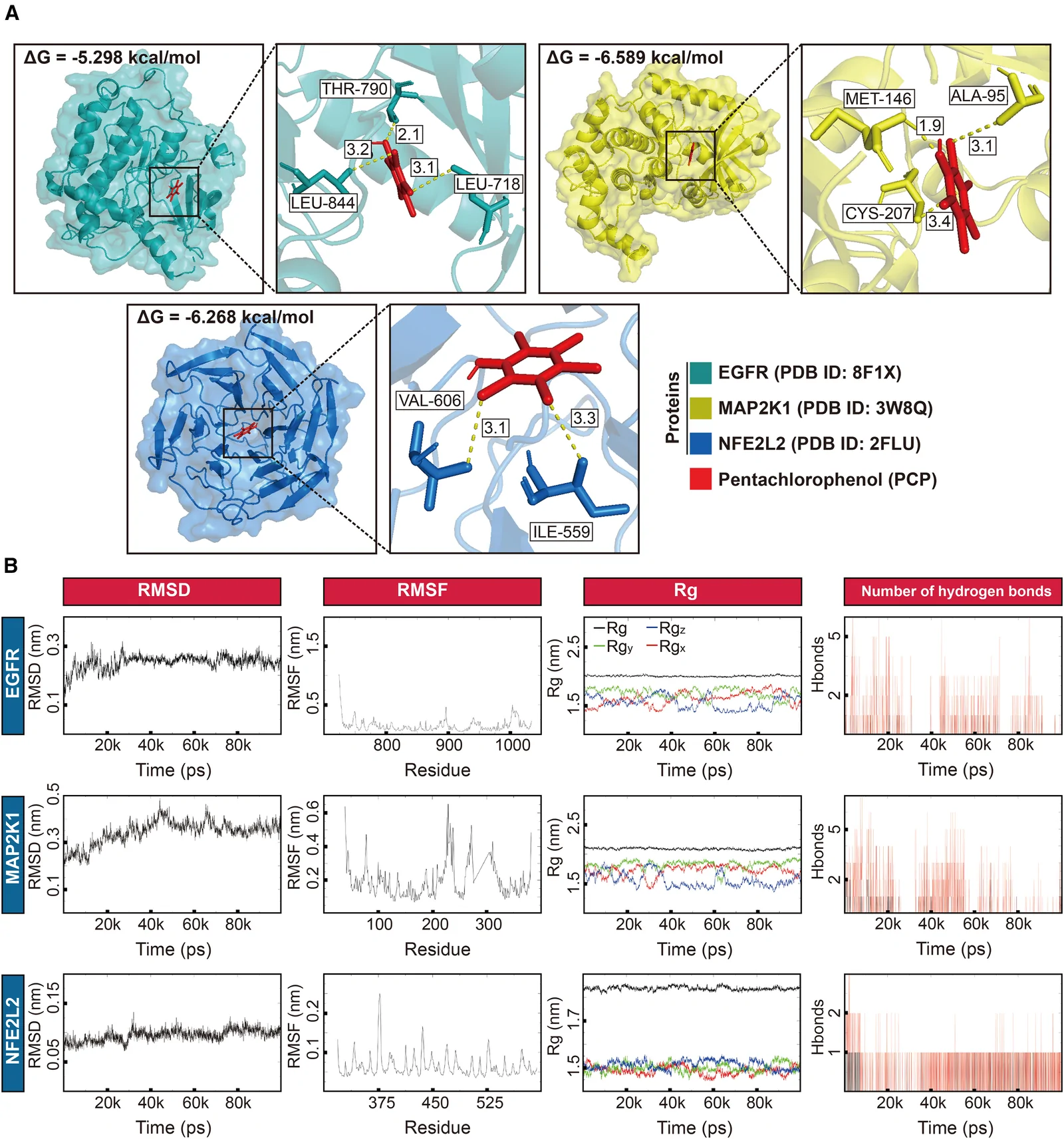
MD and MDS analyses of PCP-EGFR, PCP-MAP2K1, and PCP-NFE2L2 complexes (A) Predicted binding modes of PCP with EGFR, MAP2K1, or NFE2L2 proteins using MD. Contacting residues, intermolecular distances, and binding affinities (ΔG) are displayed. (B) The RMSD, RMSF, Rg, and number of hydrogen bonds of the predicted complexes during the 100-ns MDS. RMSD, root-mean-square deviation; RMSF, root-mean-square fluctuation; Rg, radius of gyration; MD, molecular docking; MDS, molecular dynamics simulation.

To validate the interaction between PCP and its key targets, including EGFR, MAP2K1, and NFE2L2, we conducted MD and all-atom MDS. Docking revealed favorable binding of PCP to the druggable pockets of EGFR, MAP2K1, and NFE2L2, with binding affinities of approximately −5.30, −6.59, and −6.27 kcal/mol, respectively. Key residues involved in the interactions included Thr-790, Leu-844, and Leu-718 for EGFR; Met-146, Ala-95, and Cys-207 for MAP2K1; and Val-606 and Ile-559 for NFE2L2 (Figure 5A). MDS results demonstrated stable complex dynamics, as indicated by low RMSD and RMSF values, consistent Rg, and limited solvent-accessible surface area fluctuations (Figures 5B and S7). Intermittent hydrogen bonds were maintained throughout the simulations, supporting stable binding (Figure 5B). These findings indicate that PCP binds directly and stably to EGFR, MAP2K1, and NFE2L2 proteins.

### Knockdown of MAP2K1 or EGFR, or overexpression of NFE2L2, partially suppressing PCP-induced invasiveness of BLCA cells

**Figure 6:**
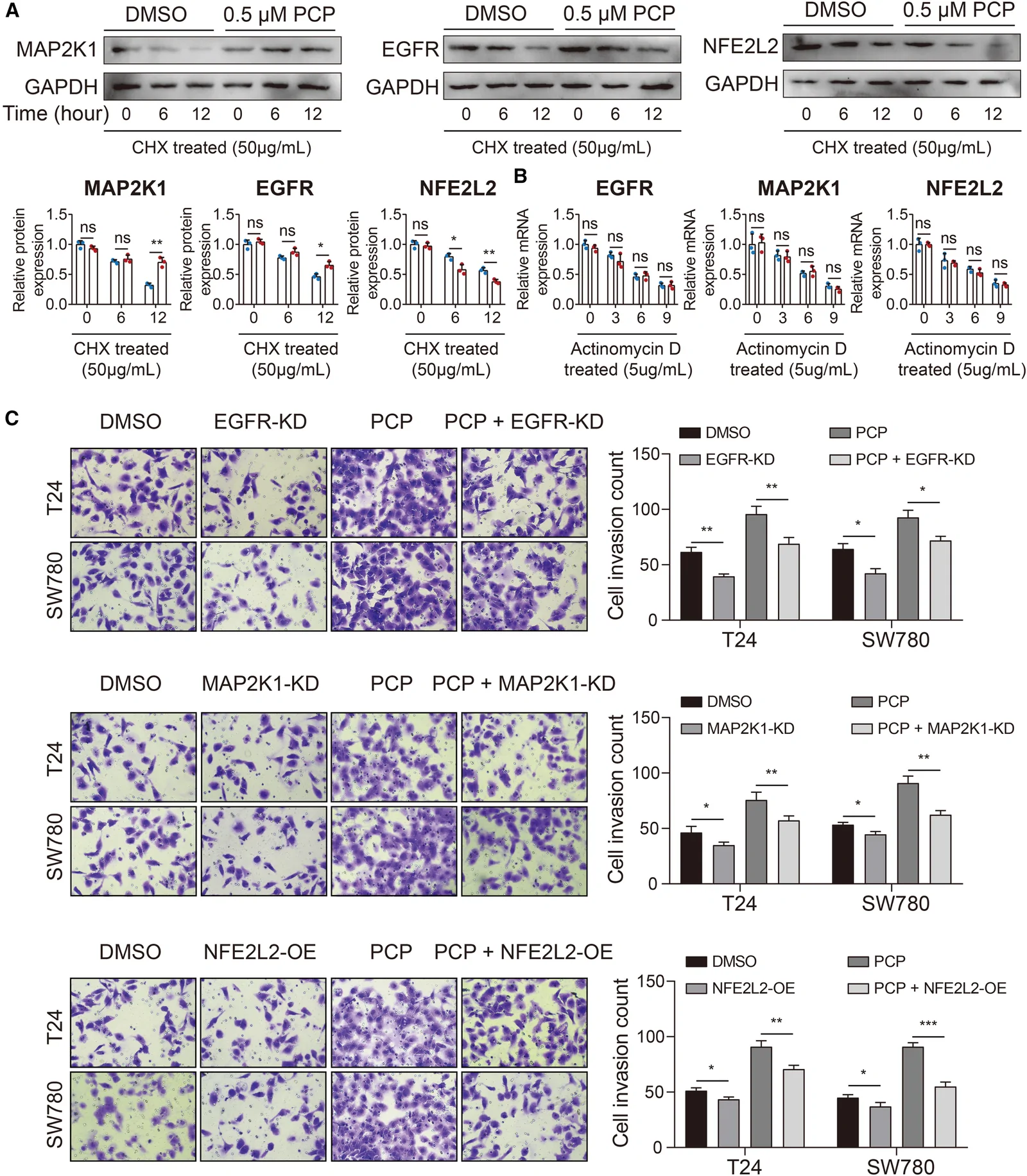
Knockdown of MAP2K1 or EGFR, or overexpression of NFE2L2, partially suppresses the PCP-induced invasiveness of T24 and SW780 cells (A and B) The influence of PCP on the protein (A) and mRNA (B) stability of MAP2K1, EGFR, and NFE2L2. (C) Cell invasiveness assessed by Transwell assays following PCP treatment under conditions of EGFR knockdown, MAP2K1 knockdown, or NFE2L2 overexpression. Values are presented as mean ± SD from at least three independent experiments (*n* = 3 biological replicates). ∗*p* < 0.05. ∗∗*p* < 0.01. ∗∗∗*p* < 0.001.

To elucidate the mechanism underlying PCP-mediated regulation of EGFR, MAP2K1, and NFE2L2, we first evaluated their protein stability in SW780 cells, which exhibited a more pronounced PCP-induced invasive phenotype compared with T24 cells (Figure 1A). Cycloheximide (CHX) chase assays revealed that 0.5 μM PCP treatment significantly extended the half-life of EGFR (*p* < 0.05) and MAP2K1 (*p* < 0.01) but shortened the half-life of NFE2L2 (*p* < 0.01, Figure 6A). In contrast, actinomycin D (ActD) chase assays demonstrated that 0.5 μM PCP had no significant effect on the mRNA stability of EGFR, MAP2K1, or NFE2L2 over a 9-h observation period (all *p* > 0.05, Figure 6B). These findings indicate that PCP regulates the expression of these three targets primarily through post-translational modulation of protein stability rather than transcriptional or post-transcriptional regulation of mRNA.

We then established EGFR-knockdown, MAP2K1-knockdown, and NFE2L2-overexpressing T24 and SW780 cells, with transfection efficiency verified by RT-qPCR (all *p* < 0.05, Figure S8). Functional validation demonstrated that knockdown of EGFR or MAP2K1, or overexpression of NFE2L2, partially reversed the promotive effect of 0.5 μM PCP on the invasiveness of BLCA cells (all *p* < 0.05, Figure 6C). To explore potential hierarchical relationships among these targets, we further assessed NFE2L2 levels in EGFR-knockdown or MAP2K1-knockdown T24 and SW780 cells, with or without 0.5 μM PCP treatment. Notably, neither EGFR knockdown nor MAP2K1 knockdown altered NFE2L2 expression under basal or PCP-exposed conditions (all *p* > 0.05, Figure S9). Collectively, these data demonstrate that EGFR, MAP2K1, and NFE2L2 contribute to PCP-induced BLCA cell invasion.

## Discussion

While PCP exposure is an established risk factor for several cancers,^14^^,^^15^^,^^16^ its potential link to BLCA, particularly at environmentally relevant concentrations, remains largely unexplored. This study therefore employed an integrated approach, combining *in vitro* assays, network toxicology, and computational simulations, to clarify the effects and mechanisms of PCP in promoting BLCA cell malignancy. Treatment of T24 and SW780 cells with PCP at concentrations (0.05, 0.5, and 5 μM) reflective of human serum levels^8^^,^^9^^,^^10^ yielded a distinct phenotypic response: concentrations of 0.5 and 5 μM significantly enhanced cellular invasion, without concomitant effects on migration or proliferation. The lowest concentration tested (0.05 μM) was devoid of any significant effect. Notably, a structurally related analog, 2,4-DCP, failed to recapitulate the pro-invasive effects of PCP at equivalent exposure concentrations, supporting that the observed phenotype is not driven by non-specific chlorophenol toxicity or generalized oxidative stress, but rather by PCP-specific interactions with its target proteins. Through network toxicology, 58 overlapping BLCA-PCP target genes were identified. A robust machine learning-based predictive model highlighted EGFR, MAP2K1, and NFE2L2 as hub genes with significant prognostic significance. Elevated EGFR or MAP2K1 and reduced NFE2L2 expression were associated with poorer OS in BLCA patients and correlated with multiple invasion-related genes. MD and MDS confirmed stable binding of PCP to these proteins. Experimentally, 0.5 μM PCP increased EGFR and MAP2K1 protein stability but decreased that of NFE2L2, without altering their mRNA stability. Functional assays confirmed that knockdown of EGFR or MAP2K1, or overexpression of NFE2L2, attenuated the PCP-induced invasive phenotype. Notably, additional experiments ruled out transcriptional cross-talk between EGFR/MAP2K1 and NFE2L2, as silencing EGFR or MAP2K1 did not affect NFE2L2 mRNA levels under either control or PCP-treated conditions. In summary, environmentally relevant concentrations of PCP promote BLCA cell invasion, likely via post-translational regulation of EGFR, MAP2K1, and NFE2L2 protein stability, providing the first evidence implicating PCP as a potential environmental risk factor for BLCA progression.

PCP, a fully chlorinated phenol, is a chemically stable and highly effective biocide widely used historically as an herbicide, wood preservative, and in China, as a molluscicide for schistosomiasis control.^24^^,^^25^ Owing to its persistence as an organic pollutant, PCP undergoes slow natural degradation, particularly in anaerobic environments like waterlogged soils or sediments, where it can accumulate over the long term.^26^ The compound absorbs strongly organic matter in soils and sediments, and its lipophilicity promotes bioaccumulation in organisms and potential biomagnification in food webs.^27^ Human exposure occurs mainly through contaminated food, water, or occupational inhalation. PCP is commonly detected in human biomonitoring studies, with measurable levels in serum and urine across the general population. A previous study has demonstrated a positive linear relationship between serum and urine PCP concentrations.^28^ Nevertheless, PCP is a persistent organic pollutant that has an extended half-life of 10–20 days. Given that POPs tend to accumulate in blood and are more reliably quantified in serum, unlike urinary levels, which are more suitable for non-persistent chemical metabolites, serum PCP is likely a more accurate biomarker of recent exposure.^29^^,^^30^ Reported serum PCP concentrations in exposed populations typically range between 0.26 and 5 μM, with a maximum level of 1.143 μM reported in a large-scale Chinese study.^8^^,^^9^^,^^10^ We therefore chose concentrations of 0.05, 0.5, and 5 μM for our experiments to cover the range of exposures documented across different population groups.

To contextualize the biological relevance of PCP-induced invasiveness, we compared the magnitude of this effect with those elicited by other established BLCA promoters. Treatment with 0.5 μM PCP, a concentration within the range of human serum levels reported in exposed populations, increased invasive cell counts by ∼1.4-fold in T24 cells and ∼1.5-fold in SW780 cells. This effect is comparable with that of 0.25% cigarette smoke extract (CSE), a well-characterized BLCA risk factor that elevates invasion by ∼1.4- to 1.5-fold in T24 and UMUC3 cells.^31^ Notably, the pro-invasive potency of PCP at environmentally relevant doses is slightly milder than that of high-dose endocrine disruptors such as 10-7 μM bisphenol A (2- to 3-fold increase in T24 and 5,637 cells)^32^ or 1 μM 4-nitrophenol (PNP, ∼2-fold increase in T24 cells).^33^ However, unlike these agents, PCP exposure is pervasive in the general population due to its persistence and bioaccumulation, suggesting that even modest enhancements of invasive capacity at physiological concentrations may translate to meaningful population-level cancer risk over long-term exposure. These comparisons underscore that PCP exerts pro-invasive effects on BLCA cells at a magnitude comparable with other established environmental BLCA risk factors, supporting its potential role in BLCA progression in exposed individuals.

While epidemiological studies link PCP exposure to increased cancer risk, the molecular mechanisms remain poorly characterized. Previous investigations by Umemura et al., Wang et al., and Thota et al. demonstrated that PCP promotes tumor progression via oxidative stress and upregulation of NF-κB, STAT3, and MAPK signaling in various models.^6^^,^^34^^,^^35^ Additional evidence also indicates PCP modulates AKT and JNK activity in pancreatic cancer contexts.^36^ Despite these insights, a comprehensive understanding of PCP’s carcinogenic activity is still lacking. To address this, our work applied an integrated approach combining network toxicology, molecular dynamics simulations, and *in vitro* validation. We show that PCP enhances the invasiveness of BLCA cells through upregulation of EGFR and MAP2K1 and suppression of NFE2L2, thereby offering a novel perspective on its pro-oncogenic mechanism.

NFE2L2, a central transcription factor regulating antioxidant response and tumorigenesis, is known to protect against chemical-induced carcinogenesis, as evidenced by increased hepatobiliary tumor incidence in Nfe2l2-deficient mice exposed to PCP.^37^^,^^38^^,^^39^ In the bladder, NFE2L2 mitigates arsenic-induced injury and suppresses tumor formation in mice,^40^ yet its role in PCP-exposed BLCA cells has been unclear. Our study reveals that PCP physically interacts with NFE2L2, decreasing its protein stability and driving an invasive phenotype in BLCA cells, thereby providing new mechanistic insight into PCP-associated urothelial carcinogenesis.

EGFR and MAP2K1 constitute a core part of the RAS-RAF-MEK-ERK pathway, which is often activated by environmental toxicants and cellular stress.^41^ As an oncogenic receptor tyrosine kinase, EGFR responds to both growth factors and environmental insults, such as wood smoke PM2.5 and inorganic arsenic, by activating downstream PI3K-AKT and ERK signaling, contributing to tissue remodeling and cancer risk in lung and sinonasal epithelium.^42^^,^^43^ In BLCA, EGFR overexpression correlates with advanced disease and poor prognosis.^44^ Similarly, MAP2K1 serves as a key signaling node in toxicogenomic networks for diverse chemicals, including plasticizers and pesticides.^45^^,^^46^ This study provides the first evidence that PCP enhances BLCA cell invasion by directly binding to and modulating the stability of EGFR and MAP2K1 proteins. As clinical-grade inhibitors for these proteins exist, our findings point to a potential therapeutic strategy for BLCA patients with PCP exposure.

While the PCPRS demonstrated robust prognostic performance across most validation cohorts, we acknowledge that its failure to reach significance in the small GSE5287 cohort (*n* = 27) highlights a limitation in generalizability when applied to very small sample sizes. This caveat should be considered when translating the signature to clinical settings, particularly in studies with limited patient numbers. However, the consistency of the meta-analysis across five independent cohorts, coupled with the functional validation showing that EGFR, MAP2K1, and NFE2L2 directly mediate PCP-driven invasiveness, supports the biological and clinical relevance of the three-gene signature. Future validation in larger, prospectively collected BLCA cohorts will be necessary to further refine its utility as a predictive biomarker for environmental exposure-associated BLCA progression.

By integrating multiple approaches, this study reveals that environmentally relevant PCP exposure specifically augments the invasiveness of BLCA cells, independent of effects on migration or proliferation. EGFR, MAP2K1, and NFE2L2 were identified as key candidate mediators of this process, though further work is needed to elucidate the relative importance of these and other BLCA-PCP target genes. These findings yield novel mechanistic evidence connecting PCP to BLCA progression, which underscores the latent health threat posed by this persistent organic pollutant.

### Limitations of the study

This study has several limitations. First, direct binding of PCP to EGFR, MAP2K1, and NFE2L2 remains computationally predicted by MD and MDS and was not validated experimentally. Second, although PCP altered the stability of these proteins, the molecular machinery underlying these changes remains undefined. Third, the prognostic analyses were conducted in public BLCA cohorts without PCP exposure information, so the three-gene signature should be interpreted as a prognostic association rather than an exposure-specific biomarker. Fourth, the experimental evidence is limited to two established BLCA cell lines under an acute exposure paradigm and may not capture tumor heterogeneity or chronic low-dose exposure effects. Fifth, the partial rescue observed after EGFR or MAP2K1 knockdown or NFE2L2 overexpression suggests that additional mediators are involved. Finally, the 2,4-DCP comparison does not exclude all nonspecific toxicological mechanisms, and further mechanistic, chronic-exposure, and *in vivo* validation is required.

## Resource availability

### Lead contact

Requests for further information and resources should be directed to and will be fulfilled by the lead contact, Cundong Liu (cundongliu@163.com).

### Materials availability

This study did not generate new unique reagents.

### Data and code availability

• Processed RNA-seq data from TCGA-BLCA and the four GEO cohorts (GSE13507, GSE31684, GSE32894, and GSE5287) have been deposited at Zenodo and are publicly available as of the date of publication at https://doi.org/10.5281/zenodo.21032951. Raw RNA-seq data from TCGA and GEO are accessible via their respective public repositories: TCGA-BLCA data are available at UCSC Xena (https://xena.ucsc.edu/, Xena ID: TCGA-BLCA), and GEO cohort data are accessible at the Gene Expression Omnibus (https://ncbi.nlm.nih.gov/geo/) under accession numbers GSE13507, GSE31684, GSE32894, and GSE5287.
• All custom R scripts have been deposited at Zenodo and are publicly available as of the date of publication at https://doi.org/10.5281/zenodo.21032951.
• Any additional information required to reanalyze the data reported in this paper is available from the lead contact upon request.

## Acknowledgments

The authors convey special thanks to the Medical Experimental Center of the Third Affiliated Hospital, 10.13039/501100010096Southern Medical University.

## Author contributions

Conceptualization, C.Y. and C.L.; data curation, R.Z., W.Z., M.X., K.Z., and T.Z.; formal analysis, R.Z., W.Z., M.X., and W.L.; methodology, R.Z., W.Z., M.X., K.Z., and T.Z.; investigation, R.Z., W.Z., M.X., W.L., and T.Z.; resources, R.Z., W.Z., and M.X.; writing – original draft, R.Z., W.Z., and M.X.; writing – review and editing, R.Z., W.Z., M.X., K.Z., W.L., T.Z., C.Y., and C.L.; project administration, C.Y. and C.L.; supervision, C.Y. and C.L. All authors read and approved the final manuscript.

## Declaration of interests

The authors declare no competing interests.

## STAR★Methods

### Key resources table

**Table:** 

REAGENT or RESOURCE	SOURCE	IDENTIFIER
**Antibodies**		

Rabbit anti-EGFR	ABclonal	Cat# A23381; RRID: AB_2863471
Rabbit anti-MAP2K1	ABclonal	Cat# A19565; RRID: AB_2863472
Rabbit anti-NFE2L2	ABclonal	Cat# A3577; RRID: AB_2863473
Rabbit anti-GAPDH	ABclonal	Cat# A19056; RRID: AB_2863474
HRP-conjugated secondary antibody	ABclonal	Cat# AS014; RRID: AB_2863475

**Chemicals, peptides, and recombinant proteins**		

Pentachlorophenol (PCP)	Merck	Cat# P2604; CAS: 87-86-5; PubChem CID: 992
2,4-Dichlorophenol (2,4-DCP)	Merck	Cat# 105953; CAS# 120-83-2; PubChem CID: 164852
Dimethyl sulfoxide (DMSO)	Merck	Cat# D2650; CAS: 67-68-5; PubChem CID: 679
Puromycin dihydrochloride	Sigma-Aldrich	Cat# P9620; CAS: 58-58-2; PubChem CID: 9548609
Actinomycin D (ActD)	Sigma-Aldrich	Cat# A9415; CAS: 50-76-0; PubChem CID: 441140
Cycloheximide (CHX)	MedChemExpress	Cat# HY-12320; CAS: 66-81-9; PubChem CID: 6167
Matrigel Growth Factor Reduced (GFR)	BD Biosciences	Cat# 354230; RRID: AB_10063508
Crystal violet	Sigma-Aldrich	Cat# C0775; CAS: 548-62-9; PubChem CID: 11056
Paraformaldehyde	Sigma-Aldrich	Cat# P6148; CAS: 30525-89-4; PubChem CID: 9859

**Critical commercial assays**		

CCK-8 assay kit	Beyotime	Cat# CK04; RRID: AB_2863476
BCA protein assay kit	Beyotime	Cat# P0012; RRID: AB_2863477
PrimeScript RT reagent kit with gDNA Eraser	Takara	Cat# RR047A; RRID: AB_2863478
TB Green Premix Ex Taq II	Takara	Cat# RR820A; RRID: AB_2863479

**Deposited data**		

TCGA-BLCA RNA-seq data	UCSC Xena	https://xena.ucsc.edu/; Xena ID: TCGA-BLCA
GEO dataset GSE13507	GEO	GSE13507
GEO dataset GSE31684	GEO	GSE31684
GEO dataset GSE5287	GEO	GSE5287
GEO dataset GSE32894	GEO	GSE32894
PDB structure EGFR (8F1X)	RCSB PDB	PDB: 8F1X
PDB structure MAP2K1 (3W8Q)	RCSB PDB	PDB: 3W8Q
PDB structure NFE2L2 (2FLU)	RCSB PDB	PDB: 2FLU

**Experimental models: Cell lines**		

Human bladder cancer cell line T24	ATCC	ATCC HTB-4; RRID: CVCL_0554
Human bladder cancer cell line SW780	ATCC	ATCC HTB-12; RRID: CVCL_0547
HEK293T cell line	ATCC	ATCC CRL-3216; RRID: CVCL_0063

**Oligonucleotides**		

EGFR qPCR primer Forward	Tsingke Biotechnology	5′-AGGCACGAGTAACAAGCTCAC-3′
EGFR qPCR primer Reverse	Tsingke Biotechnology	5′-ATGAGGACATAACCAGCCACC-3′
MAP2K1 qPCR primer Forward	Tsingke Biotechnology	5′-CAATGGCGGTGTGGTGTTC-3′
MAP2K1 qPCR primer Reverse	Tsingke Biotechnology	5′-GATTGCGGGTTTGATCTCCAG-3′
NFE2L2 qPCR primer Forward	Tsingke Biotechnology	5′-TCAGCGACGGAAAGAGTATGA-3′
NFE2L2 qPCR primer Reverse	Tsingke Biotechnology	5′-CCACTGGTTTCTGACTGGATGT-3′
GAPDH qPCR primer Forward	Tsingke Biotechnology	5′-GGAGCGAGATCCCTCCAAAAT-3′
GAPDH qPCR primer Reverse	Tsingke Biotechnology	5′-GGCTGTTGTCATACTTCTCATGG-3′
shRNA targeting EGFR sense	Tsingke Biotechnology	5′-AGAATGTGGAATACCTAAGG-3′
shRNA targeting EGFR antisense	Tsingke Biotechnology	5′-CCTTAGGTATTCCACATTCT-3′
shRNA targeting MAP2K1 sense	Tsingke Biotechnology	5′-GCTTCTATGGTGCGTTCTACA-3′
shRNA targeting MAP2K1 antisense	Tsingke Biotechnology	5′-TGTAGAACGCACCATAGAAGC-3′

**Recombinant DNA**		

pLKO.1-puro lentiviral vector	Addgene	Addgene #8453; RRID: Addgene_8453
pLKO.1-shEGFR	Tsingke Biotechnology	N/A
pLKO.1-shMAP2K1	Tsingke Biotechnology	N/A
Lentiviral NFE2L2 overexpression vector (NM_006164)	Tsingke Biotechnology	N/A

**Software and algorithms**		

R v4.4.2	R Project	https://www.r-project.org/; RRID: SCR_001905
GraphPad Prism v8.0.1	GraphPad Software	https://www.graphpad.com/; RRID: SCR_002798
ImageJ	NIH	https://imagej.nih.gov/ij/; RRID: SCR_003070
PyMOL v3.0.3	Schrödinger	https://pymol.org/; RRID: SCR_000305
AutoDockTools v1.5.6	Scripps Research	https://autodocksuite.scripps.edu/; RRID: SCR_000050
AutoDock Vina v1.2.5	Scripps Research	https://vina.scripps.edu/; RRID: SCR_011095
GROMACS v2022.4	GROMACS Development Team	https://www.gromacs.org/; RRID: SCR_014565
Cytoscape v3.8.0	Cytoscape Consortium	https://cytoscape.org/; RRID: SCR_003032
OpenBabel v3.x	OpenBabel	http://openbabel.org/; RRID: SCR_007519

**Other**		

Lipofectamine 3000 Transfection Reagent	Thermo Fisher Scientific	Cat# L3000015; RRID: AB_2621642
Transwell inserts (8 μm polycarbonate)	Corning	Cat# 3422; RRID: AB_2863480
PVDF membranes (0.45 μm)	Millipore	Cat# IPVH00010; RRID: AB_2863481
TRIzol Reagent	Thermo Fisher Scientific	Cat# 15596026; RRID: AB_2863482
RPMI-1640 Medium	Gibco (Thermo Fisher)	Cat# 11875093; RRID: AB_2863483
Leibovitz’s L-15 Medium	Merck	Cat# L1518; RRID: AB_2863484
Fetal bovine serum (FBS)	Hyclone (Cytiva)	Cat# SH30084.03; RRID: AB_2863485
Penicillin-Streptomycin (10,000 U/mL)	Invitrogen (Thermo Fisher)	Cat# 15140122; RRID: AB_2863486
0.25% Trypsin-EDTA	Gibco (Thermo Fisher)	Cat# 25200056; RRID: AB_2863487
ECL Western Blotting Detection Reagent	Beyotime	Cat# P0018; RRID: AB_2863488

### Experimental model and study participant details

#### Cell lines

Human BLCA cell lines T24 and SW780 were obtained from the American Type Culture Collection (ATCC, USA). T24 cells were cultured in RPMI-1640 medium (Gibco, USA) supplemented with 10% fetal bovine serum (FBS; Hyclone, USA) and 1% penicillin–streptomycin (Invitrogen, USA). SW780 cells were cultured in Leibovitz’s L-15 medium (Merck, USA) supplemented with 10% FBS and 1% penicillin–streptomycin. Both cell lines were maintained at 37°C in a humidified incubator with 5% CO_2_. Cells were passaged using 0.25% trypsin–EDTA, routinely tested for mycoplasma contamination, and used in the logarithmic growth phase. Cell viability was confirmed by trypan blue exclusion (>95%) prior to experiments.

### Method details

#### Chemical exposure

PCP (CAS 87-86-5, Merck, Germany) and 2,4-DCP (CAS 120-83-2, Merck, Germany) were dissolved in dimethyl sulfoxide (DMSO; Cat# D2650, Merck) and diluted to final working concentrations of 0.05, 0.5, and 5 μM in serum-free medium. Cells were exposed to the indicated concentrations for 24 h; control groups received an equivalent volume of DMSO. Following treatment, cells were washed twice with phosphate-buffered saline (PBS), dissociated with 0.25% trypsin–EDTA, and processed for downstream assays. For invasion and migration assays, the same drug concentrations were maintained in both upper and lower chambers. The final DMSO concentration was maintained at 0.1% (v/v) across all PCP-treated and vehicle-control groups.

#### Lentivirus production

Lentiviral shRNA vectors targeting EGFRand MAP2K1, as well as a lentiviral overexpression vector for NFE2L2, were constructed by Tsingke Biotechnology (China). The shRNAs were cloned into the pLKO.1 lentiviral backbone. Target sequences were: EGFR sense 5′-AGAATGTGGAATACCTAAGG-3′ and antisense 5′-CCTTAGGTATTCCACATTCT-3’; MAP2K1 sense 5′-GCTTCTATGGTGCGTTCTACA-3′ and antisense 5′-TGTAGAACGCACCATAGAAGC-3’. For NFE2L2overexpression, the full-length human coding sequence (NM_006164) was subcloned into a lentiviral expression vector. Corresponding empty vectors served as controls. Lentiviral particles were produced by co-transfecting HEK293T cells with transfer plasmids and packaging/envelope plasmids using Lipofectamine 3000 (Thermo Fisher Scientific, USA) according to the manufacturer’s instructions. At 48 h post-transfection, viral supernatants were collected, centrifuged at 1000×g for 5 min, and used to infect T24 and SW780 cells at a multiplicity of infection (MOI) of 10. Infected cells were selected with 2 μg/mL puromycin for 7 days to establish stable knockdown or overexpression pools. Knockdown and overexpression efficiency were validated by RT–qPCR.

#### Transwell invasion assay

Transwell inserts with 8-μm polycarbonate membranes (Corning, USA) were coated with Matrigel (1:8 in serum-free medium; BD Biosciences, USA) and allowed to solidify at 37°C for 1 h. A total of 5 × 10^4^ cells in serum-free medium were seeded into the upper chambers, and 600 μL of medium containing 10% FBS was added to the lower chambers as a chemoattractant. After 24 h, non-invading cells on the upper surface were removed with a cotton swab. Invaded cells on the underside were fixed with 4% paraformaldehyde, stained with 0.1% crystal violet, and counted in five random fields per insert under an inverted microscope (Nikon, Japan).

#### Wound healing assay

Cells were seeded in 6-well plates at 1 × 10^6^ cells/well and cultured to ∼90% confluence. A linear scratch was made using a sterile 200-μL pipette tip. Detached cells were removed by gentle PBS washing, and cells were cultured in serum-free medium. Wound closure was imaged at 0 and 24 h, and migration distance was quantified using ImageJ.

#### CCK-8 proliferation assay

Cells were seeded in 96-well plates at 3 × 10^3^ cells/well and incubated for 12–72 h. At each time point, 10 μL of CCK-8 reagent (Beyotime, China) was added and incubated for 2 h. Absorbance was measured at 450 nm using a BioTek microplate reader (USA). Proliferation was expressed as absorbance values normalized to the untreated control group.

#### Western blot

Total protein was extracted using RIPA lysis buffer (Beyotime, China) containing protease inhibitors (Roche, USA). Protein concentration was determined by BCA assay (Beyotime). Equal amounts of protein were separated by 10% SDS–PAGE and transferred onto PVDF membranes (Millipore, USA). Membranes were blocked with 5% non-fat milk in TBST for 1 h at room temperature, then incubated overnight at 4°C with primary antibodies: anti-EGFR (1:1000, Cat# A23381, ABclonal), anti-MAP2K1 (1:1000, Cat# A19565, ABclonal), anti-NFE2L2 (1:1000, Cat# A3577, ABclonal), and anti-GAPDH (1:50 000, Cat# A19056, ABclonal). After washing, membranes were incubated with HRP-conjugated secondary antibody (1:5000, Cat# AS014, ABclonal) for 1 h at room temperature. Protein bands were visualized using an ECL detection system (Beyotime) and quantified with ImageJ.

#### RT–qPCR

Total RNA was extracted with TRIzol reagent (Thermo Fisher Scientific, USA) and treated with DNase I (Roche, Switzerland) to remove genomic DNA. RNA quality was assessed by spectrophotometry and agarose gel electrophoresis. cDNA was synthesized from 1 μg RNA using the PrimeScript RT kit (Takara, Japan). RT–qPCR was performed with TB Green Premix Ex Taq II (Takara) on an Applied Biosystems QuantStudio 1 system (Thermo Fisher Scientific) using the following cycling conditions: 95°C for 30 s; 40 cycles of 95°C for 5 s and 60°C for 30 s; followed by a melt curve (65°C–95°C). Primers were obtained from PrimerBank and synthesized by Tsingke Biotechnology. Sequences were: EGFR: F: 5′-AGGCACGAGTAACAAGCTCAC-3′, R: 5′-ATGAGGACATAACCAGCCACC-3’; MAP2K1: F: 5′-CAATGGCGGTGTGGTGTTC-3′, R: 5′-GATTGCGGGTTTGATCTCCAG-3’; NFE2L2: F: 5′-TCAGCGACGGAAAGAGTATGA-3′, R: 5′-CCACTGGTTTCTGACTGGATGT-3’; GAPDH: F: 5′-GGAGCGAGATCCCTCCAAAAT-3′, R: 5′-GGCTGTTGTCATACTTCTCATGG-3’.

Relative mRNA expression was calculated using the 2^−ΔΔCt^ method with GAPDH as the reference gene.

#### Transcription and protein stability

For transcription inhibition, SW780 cells were pretreated with 5 μg/mL ActD (Sigma-Aldrich, USA) for 1 h, followed by 9 h exposure to 0.5 μM PCP or DMSO. Cells were then harvested for RT–qPCR measurement of EGFR, MAP2K1, and NFE2L2 mRNA. For protein stability, SW780 cells were treated with 0.5 μM PCP or DMSO for 24 h, after which 50 μg/mL CHX (MedChemExpress, USA) was added to block protein synthesis. Cells were harvested at 0, 6, and 12 h post-CHX, and protein levels were measured by western blot.

#### Bioinformatics analysis

RNA-seq data for TCGA-BLCA were downloaded from UCSC Xena, together with follow-up duration and survival status. Four additional BLCA cohorts, including GSE13507, GSE31684, GSE5287, and GSE32894, were obtained from the Gene Expression Omnibus (GEO), and OS information was retrieved from GEO or the original publications.

Data were normalized using R v4.4.2. Genes with average expression <0.5 and cases with follow-up ≤30 days were excluded. TCGA RNA-seq data were transformed to log2 (TPM +1), and microarray data to log2(expression +1). Batch effects between TCGA and GEO datasets were corrected using the ComBat algorithm (sva package).

Potential PCP targets were collected from TargetNet, SwissTargetPrediction, ChEMBL, and STITCH, merged, and deduplicated. BLCA-associated genes were retrieved from GeneCards, OMIM, and TTD, and further refined by differential-expression analysis (|log_2_FC| > 1, FDR <0.05) and univariate Cox regression (FDR <0.05) in TCGA-BLCA. Protein–protein interaction networks were constructed using STRING (combined-score threshold ≥0.4) and visualized in Cytoscape v3.8.0; hub genes were identified with the cytoHubba plugin.

#### Prognostic-model construction

To evaluate the prognostic value of PCP-related genes, 101 machine-learning-algorithm combinations were evaluated using the Mime R package. Algorithms included Random Survival Forest (RSF), Elastic Net (Enet), Stepwise Cox (StepCox), CoxBoost, plsRcox, SuperPC, GBM, SurvivalSVM, Ridge, and Lasso, paired with Lasso, StepCox, CoxBoost, or RSF variable-selection filters. TCGA-BLCA served as the training cohort; GSE13507, GSE31684, GSE5287, and GSE32894 served as external-validation cohorts. 10-fold cross-validation and 1000 bootstrap resamples (rms package) were used to correct for overfitting. The final model was selected based on the highest mean C-index in the validation cohorts and used to calculate a PCPRS for each patient.

#### MD

MD was performed using PyMOL v3.0.3, AutoDockTools v1.5.6, and AutoDock Vina v1.2.5. Crystal structures were obtained from RCSB PDB: EGFR (8F1X), MAP2K1 (3W8Q), and NFE2L2 (2FLU). Receptors were prepared by removing co-crystallized ligands and nonessential heteroatoms, retaining catalytic ions, adding polar hydrogens, and assigning Gasteiger charges; receptors were kept rigid and ligands were treated as flexible. PCP was geometry-optimized with the MMFF94 force field in OpenBabel and converted to PDBQT in AutoDockTools. The docking grid box (28 × 28 × 28 Å^3^) was centered on the native ligand. Vina was run with exhaustiveness = 120 and default iterated global–local search with BFGS local optimization. Ten independent runs with different random seeds were performed per complex; poses were ranked by predicted binding affinity (kcal/mol), and the lowest-energy pose was selected for analysis. Protein–ligand contacts were annotated in PyMOL.

#### MDS

MDS was performed using GROMACS v2022.4. Hydrogen atoms were added with pdb2gmx using the AMBER99SB-ILDN force field; protonation states were adjusted to pH 7.4 using PROPKA. Ligand topologies were generated with the CHARMM General Force Field via SwissParam and validated by energy minimization. Systems were solved in cubic boxes with TIP3P water extending ≥1.0 nm from the solute, neutralized with Na^+^/Cl^−^ at 0.15 M, and energy-minimized by steepest descent. Equilibration consisted of 100-ps NVT (V-rescale thermostat, τ_T = 0.1 ps, 310 K) and 200-ps NPT (Parrinello–Rahman barostat, τ_P = 2.0 ps, 1 atm). Production runs were performed for 100 ns with a 2-fs timestep; bonds involving hydrogen were constrained with LINCS. Long-range electrostatics used PME (Fourier spacing 0.12 nm, Coulomb cutoff 1.4 nm) and van der Waals interactions were truncated at 1.4 nm. RMSD, RMSF, Rg, SASA, and hydrogen-bond occupancy were computed with GROMACS built-in tools.

More detailed methodology has been added to supplemental information.

### Quantification and statistical analysis

All statistical analyses were performed using R v4.4.2 and GraphPad Prism v8.0.1. Continuous variables are expressed as mean ± standard deviation (SD). Group comparisons used Welch’s *t* test or Mann–Whitney U test, as appropriate; categorical variables were compared by χ^2^ or Fisher’s exact test. Spearman correlation was performed with the cor.test function in R. Principal-component analysis (PCA) used the prcomp function. Kaplan–Meier survival analysis with log rank testing was performed using the survival R package. Meta-analyses of hazard ratios were conducted using the meta R package; Cochran’s Q test determined the use of fixed- or random-effects models (substantial heterogeneity defined as *p* < 0.10). A two-sided *p* < 0.05 was considered statistically significant unless otherwise stated.

### Additional resources

TargetNet: http://targetnet.scbdd.com/.

SwissTargetPrediction: http://www.swisstargetprediction.ch/.

ChEMBL: https://www.ebi.ac.uk/chembl/.

STITCH: https://stitch.embl.de/.

STRING: https://cn.string-db.org/.

Cytoscape: https://cytoscape.org/.

GEPIA: http://gepia.cancer-pku.cn/.

ADMETLAB 3.0: https://admetlab3.scbdd.com/.

ProTox3: https://tox.charite.de/protox3/#.

ChemBlink: https://www.chemblink.com.

Human Protein Atlas: https://www.proteinatlas.org/.

RCSB PDB: https://www.rcsb.org/.

UCSC Xena: https://xena.ucsc.edu/.

GEO: https://ncbi.nlm.nih.gov/geo/.

Metascape: https://metascape.org/.

## Supplemental information

Document S1. Figures S1–S9, supplemental methods, the R code, and Tables S5–S7

Table S1. The target genes of PCP

Table S2. The BLCA-related genes identified by GeneCards, OMIM, and TTD

Table S3. The differentially-expressed genes between paracarcinoma and tumor tissues in the TCGA-BLCA cohort

Table S4. 367 of 8,600 differentially-expressed genes were associated with OS in the TCGA-BLCA cohort

Table S8. The top 100 genes are mostly associated with EGFR, MAP2K1, and NFE2L2 in BLCA
