# Supplementary material for: Pentachlorophenol enhances bladder cancer cell invasion through altered protein stability
Source: iScience. 2026 Aug 26;29(9):117357. doi: 10.1016/j.isci.2026.117357 (PMC13543803; doi:10.1016/j.isci.2026.117357)
Supplement: Supplementary file 1 [file mmc1.pdf]

iScience, Volume *29*

Supplemental information

Pentachlorophenol enhances bladder cancer cell invasion through altered protein stability

Ranran Zhou, Wansong Zhang, Ming Xia, Ker Zhang, Wei Luo, Tianhang Zhu, Cheng Yang, and Cundong Liu

1. Supplementary Figures

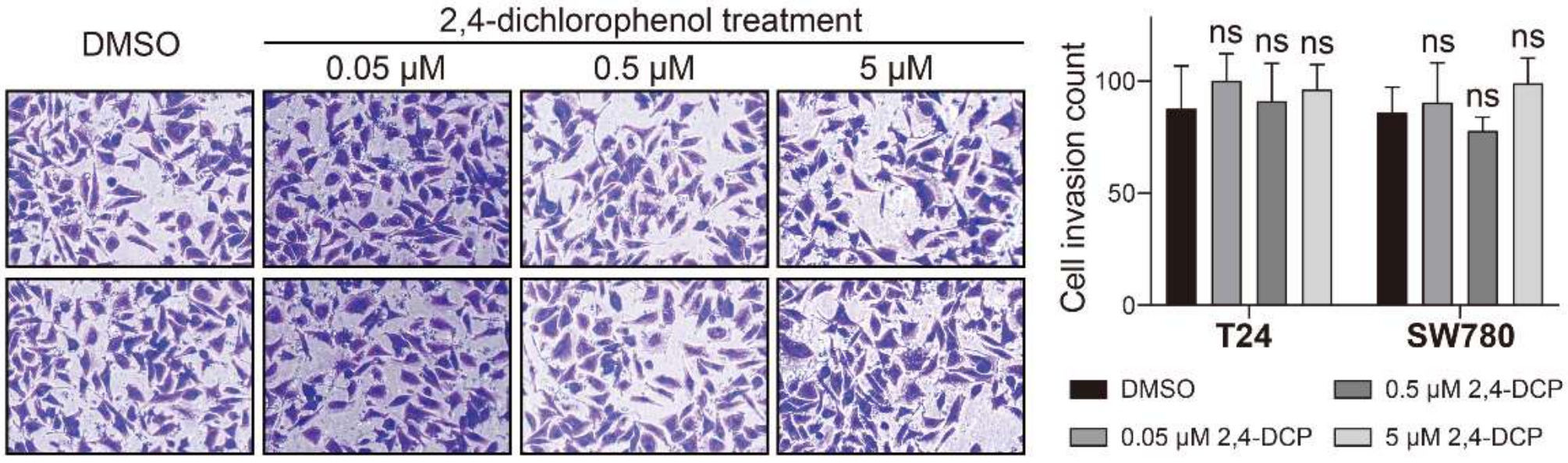

Fig. S1. 2,4-Dichlorophenol (2,4-DCP) does not affect BLCA cell invasion. T24 and SW780 cells were treated with 0.05, 0.5, or 5 μM 2,4-DCP for 24 h. (Left) Representative images of invaded cells. (Right) Quantitative analysis of invaded cell counts. *Values are presented as mean ± SD from at least three independent experiments (n = 3 biological replicates). ns, not significant.*

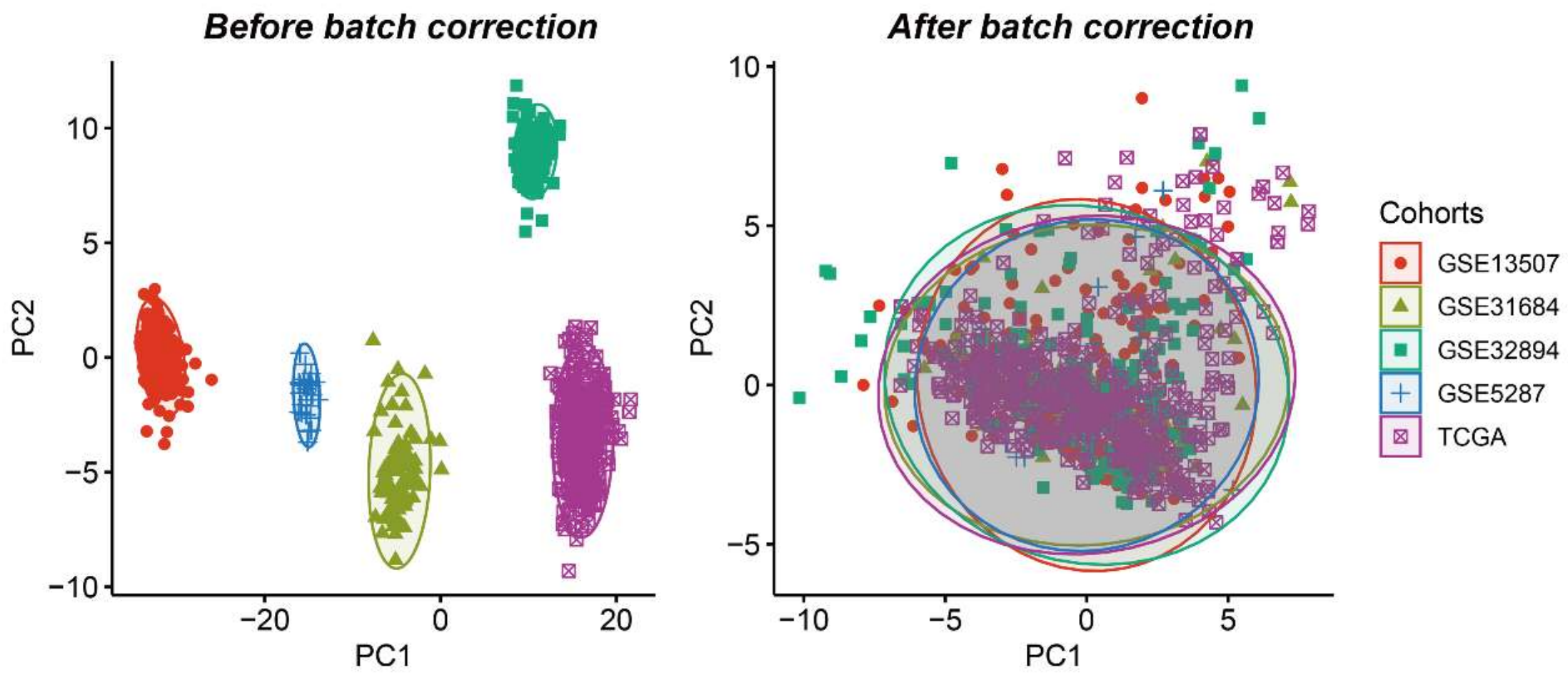

Fig. S2. Evaluating batch effect correction with principal component analysis.

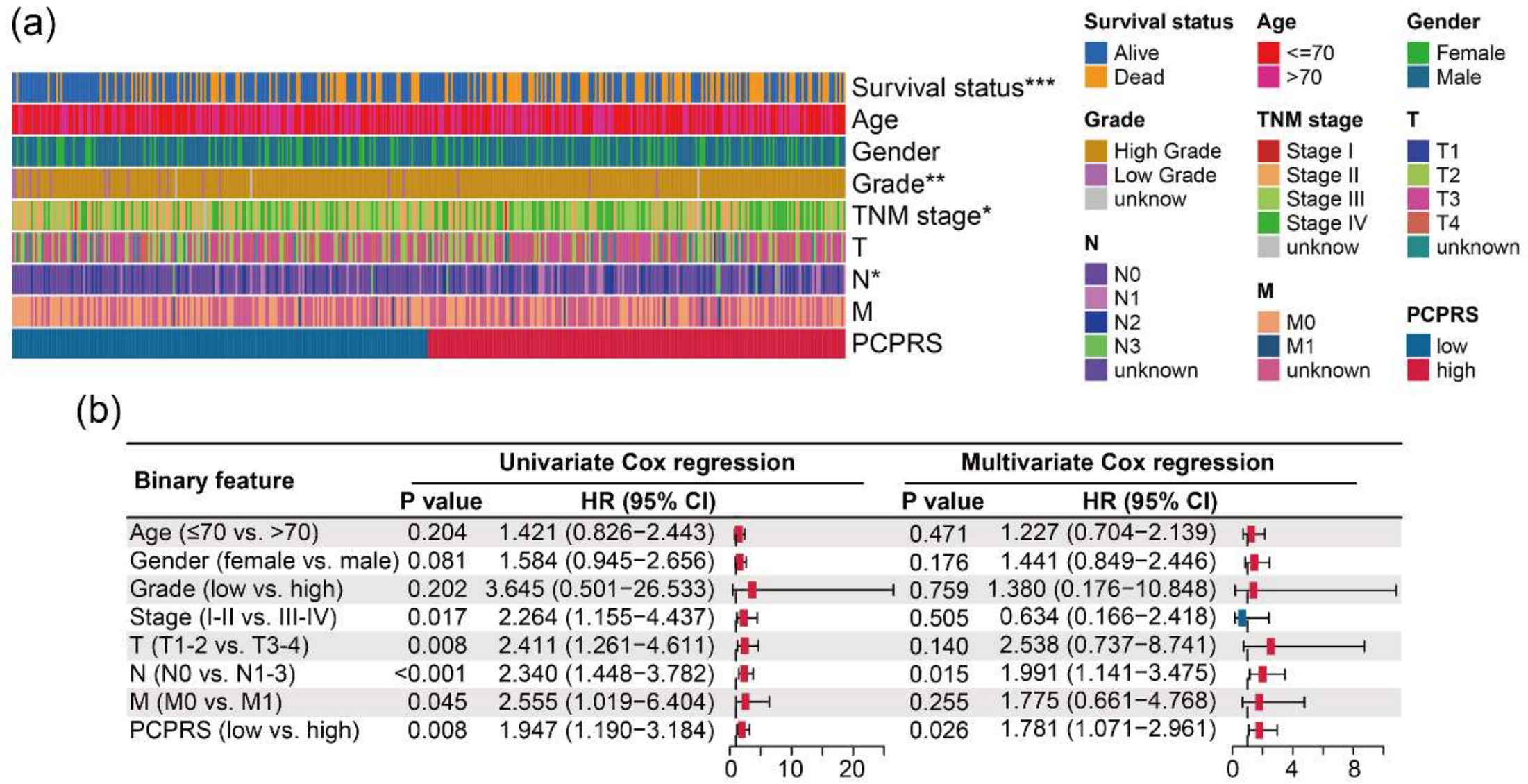

Fig. S3. PCPRS demonstrates superior performance over conventional clinicopathological parameters for predicting OS in BLCA patients. **a** The association between PCPRS and routine clinicopathological features. **b** Univariable and multivariable Cox regression analyses of OS in the TCGA-BLCA cohort. The analyses assessed the association of OS with clinicopathological features and the PCPRS. All variables were dichotomized for analysis; the optimal cut-off value for age was determined using X-tile software. *PCPRS, pentachlorophenol-related score; BLCA, bladder cancer; OS, overall survival. * P<0.05. ** P<0.01. *** P<0.001.*

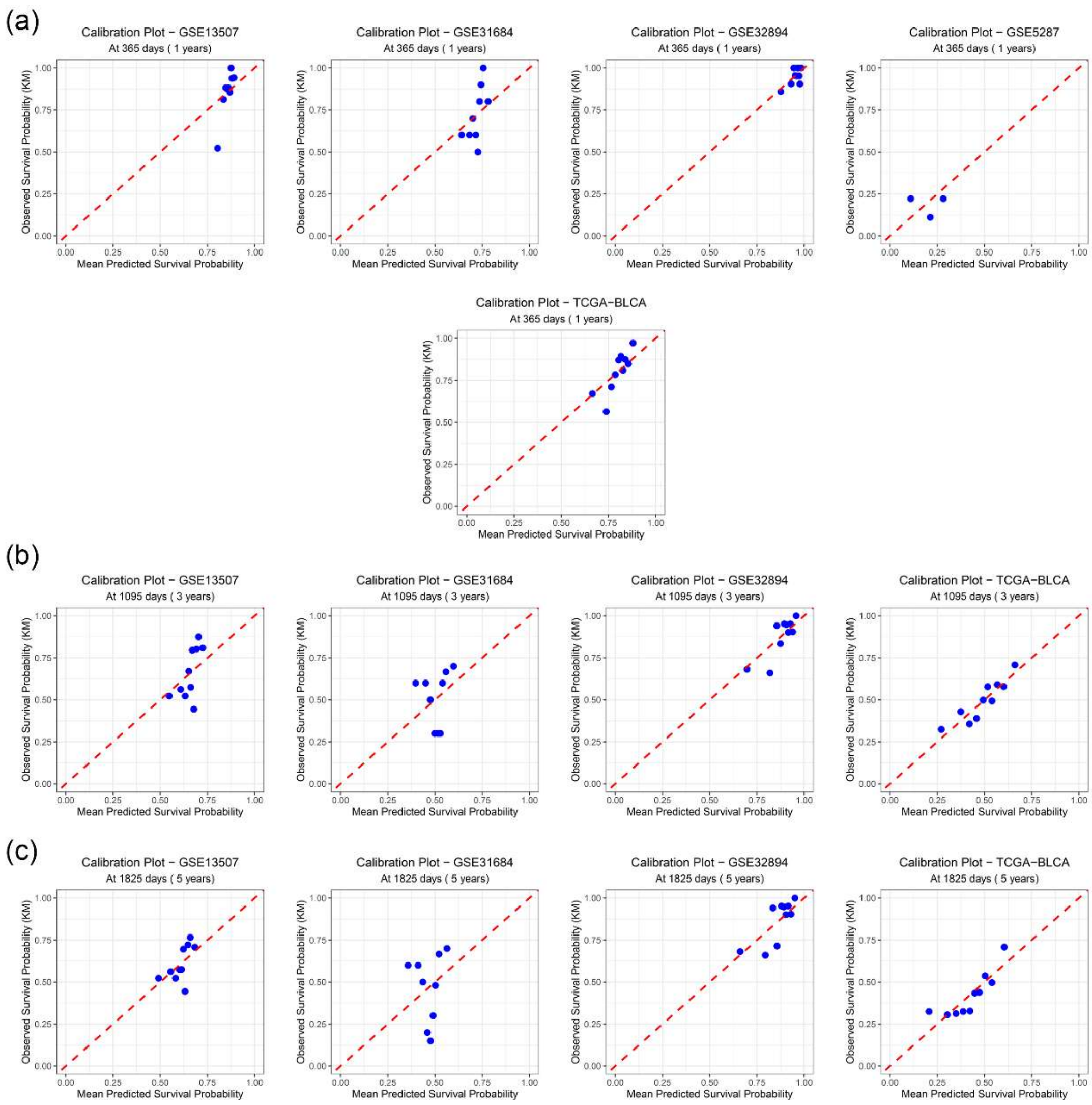

Fig. S4. Calibration plots for the PCPRS prognostic model across BLCA cohorts. Calibration curves for 1-year (**a**), 3-year (**b**), and 5-year (**c**) overall survival are shown for the TCGA-BLCA, GSE13507, GSE31684, and GSE32894 cohorts. For the GSE5287 cohort (maximum follow-up: 420 days), only the 1-year OS calibration plot is presented.

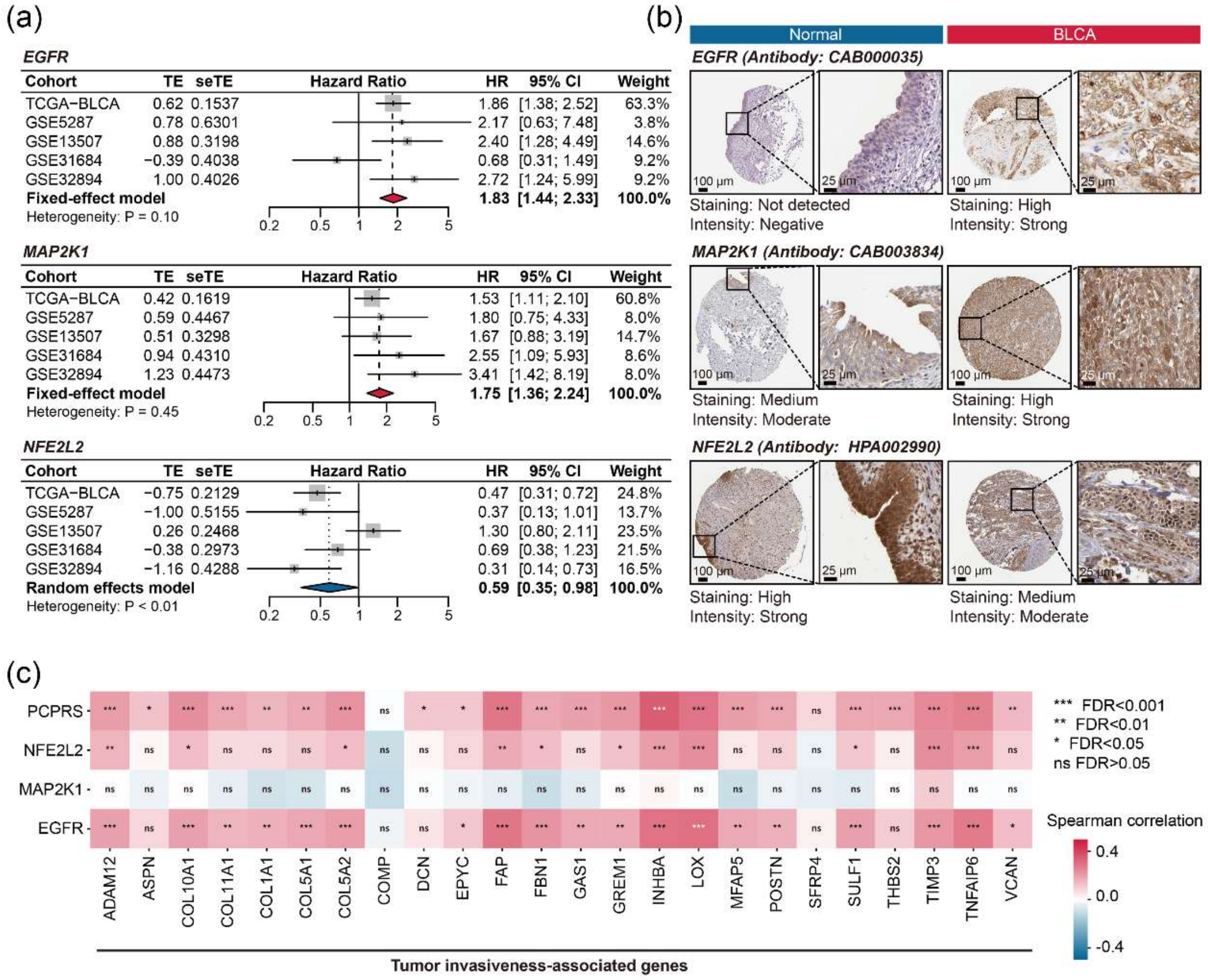

Fig. S5. High expressions of EGFR and MAP2K1 and low expression of NFE2L2 were associated with unfavorable prognosis in BLCA. **a** Meta-analyses confirmed the predictive ability of EGFR, MAP2K1, and NFE2L2 expressions to OS across TCGA-BLCA, GSE5287, GSE13507, GSE31684, and GSE32894 cohorts. **b** Protein expression levels of EGFR, MAP2K1, and NFE2L2 in normal bladder and BLCA tissues, as assessed using data from the HPA. **c** Association analysis between EGFR, MAP2K1, and NFE2L2 expression and 24 tumor invasiveness-associated genes in the TCGA-BLCA cohort. *HPA, Human Protein Atlas; * P<0.05; ** P<0.01; ***P<0.001.*

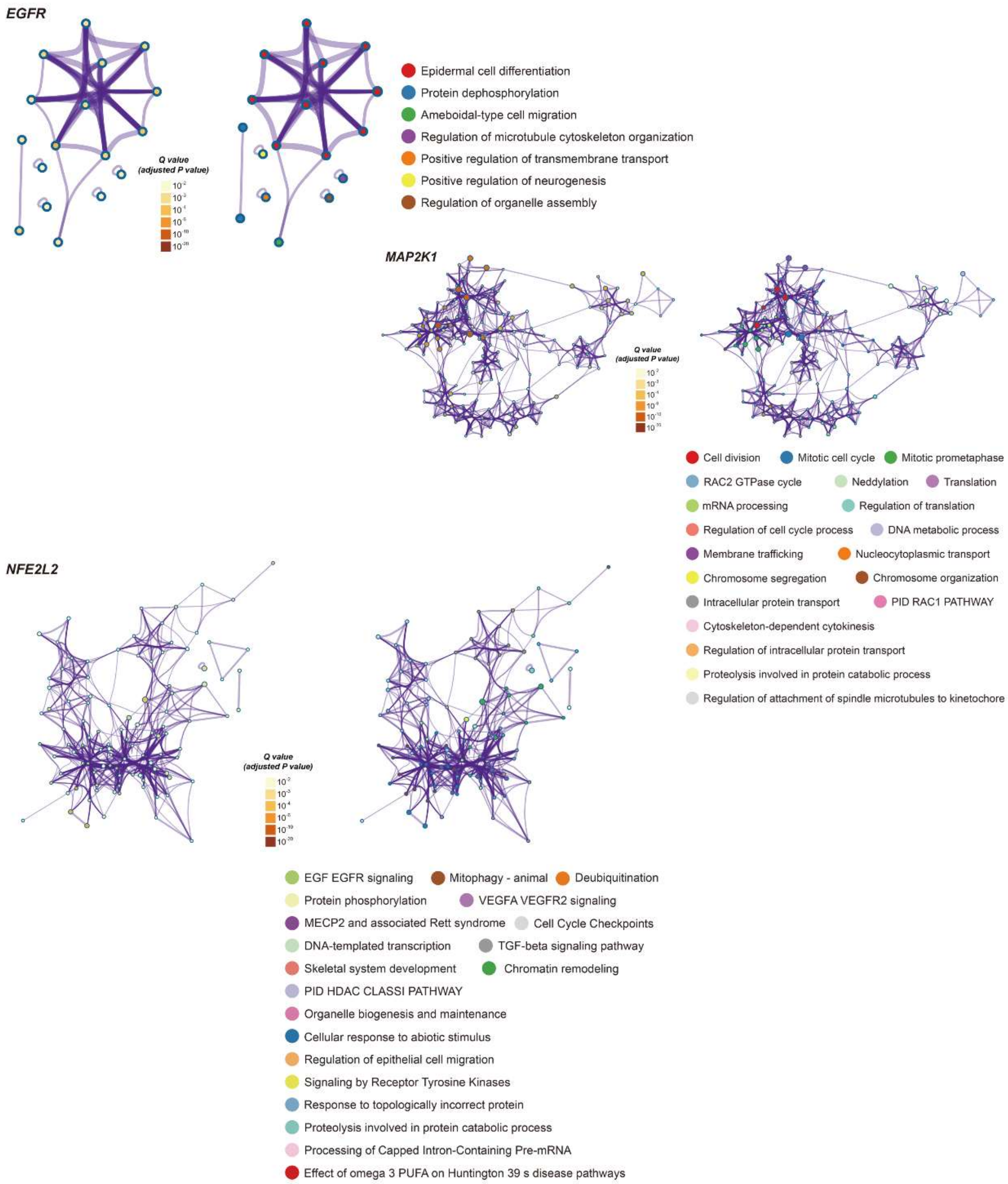

Fig. S6. The functional enrichment of Top 100 genes associated with EGFR, MAP2K1, or NFE2L2 in BLCA. The Top 100 genes associated with EGFR were identified using the GEPIA (http://gepia.cancer-pku.cn/), and the functional enrichment was conducted using the Metascape (https://metascape.org/gp/index.html#/main/step1).

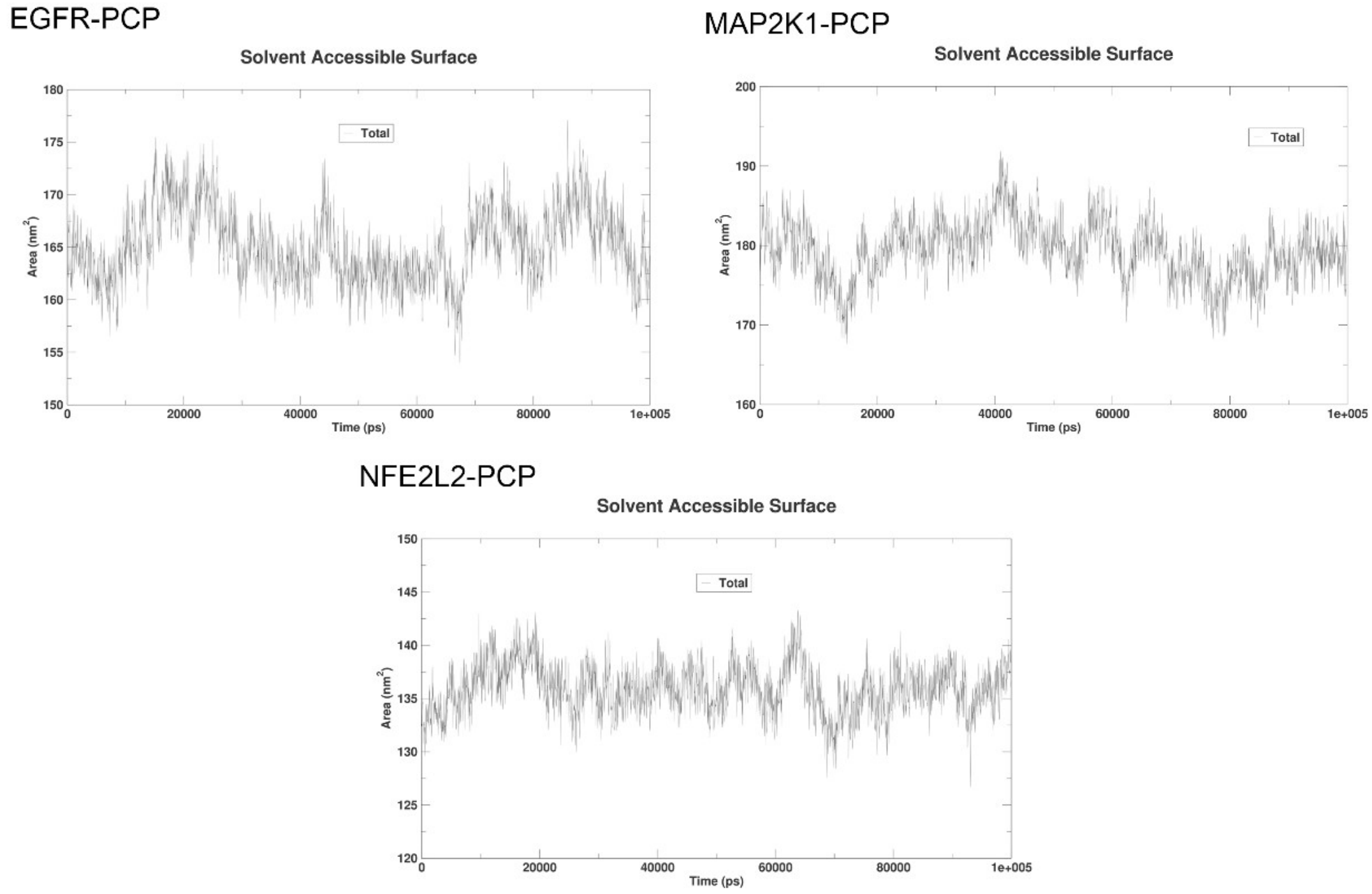

Fig. S7. Time series of SASA from molecular dynamics simulations of EGFR-PCP, MAP2K1-PCP, and NFE2L2-PCP complexes. *SASA, solvent-accessible surface area.*

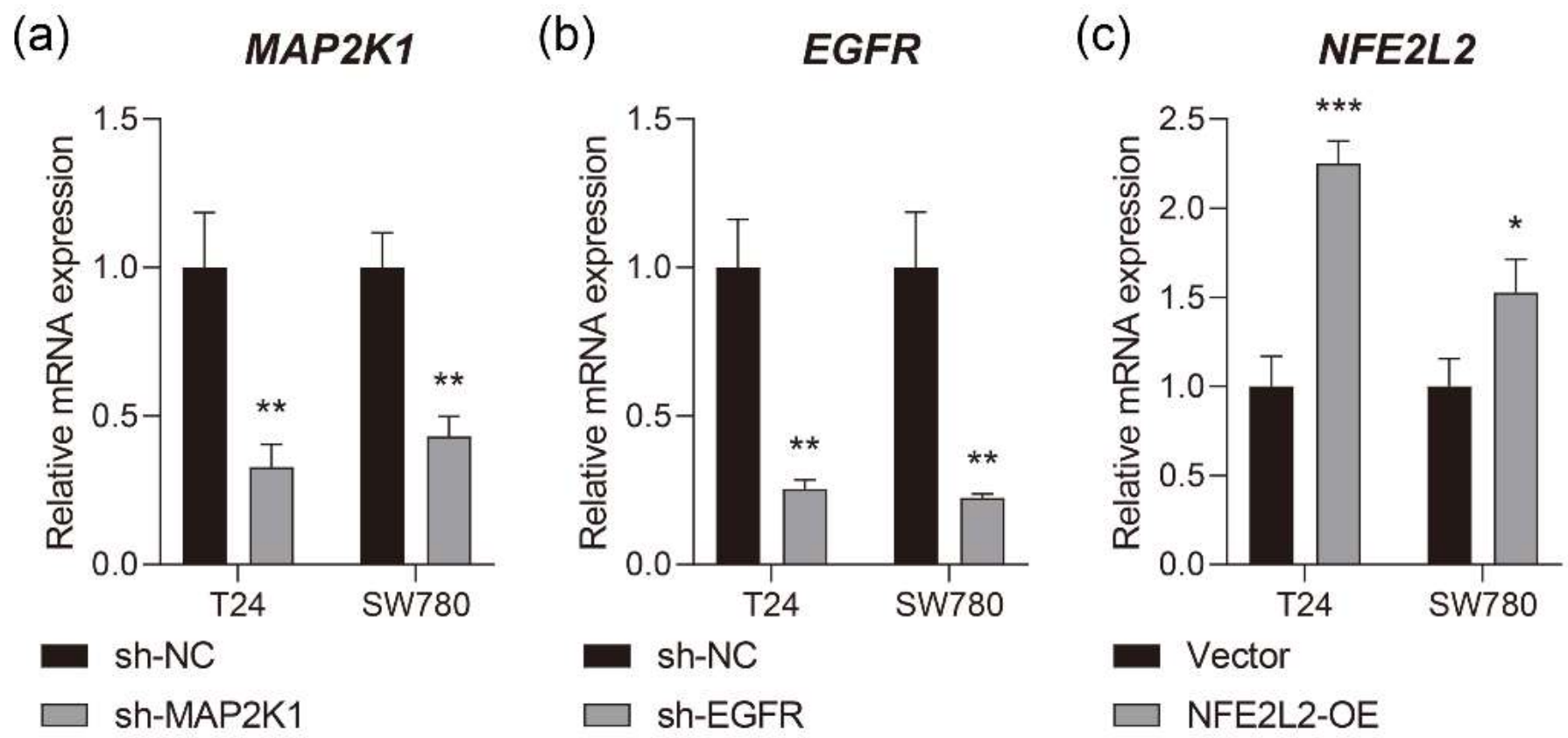

Fig. S8. Validation of transfection efficacy by RT-qPCR. The efficacy of MAP2K1 knockdown (**a**), EGFR knockdown (**b**), and NFE2L2 overexpression (**c**) was validated

using RT-qPCR in T24 and SW780 cells. *Values are presented as mean ± SD from at least three independent experiments (n = 3 biological replicates). *P<0.05; **P<0.01; ***P<0.001.*

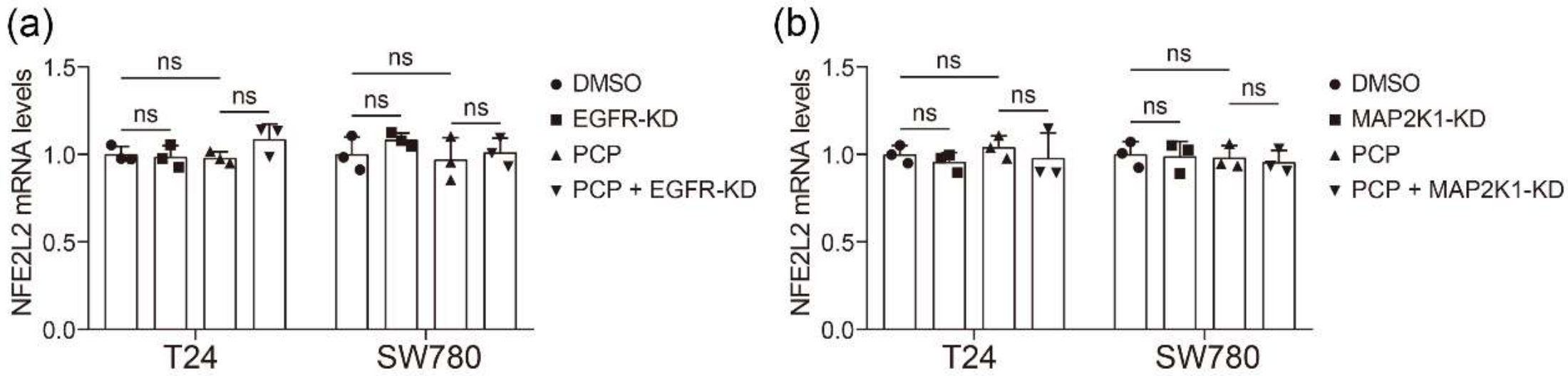

Fig. S9. NFE2L2 mRNA levels in EGFR-knockdown (**a**) or MAP2K1-knockdown (**b**) T24 and SW780 cells, with or without 0.5μM PCP treatment. *Values are presented as mean ± SD from at least three independent experiments (n = 3 biological replicates). ns, not significant.*

2. Detailed materials and methods

Materials

Pentachlorophenol (PCP, CAS 87-86-5, Cat# P2604) and 2,4-dichlorophenol (2,4-DCP; CAS# 120-83-2) were purchased from Merck (Germany). All working concentrations of PCP/2,4-DCP for cell exposure experiments were prepared in dimethylsulfoxide (DMSO; Cat# D2650, Merck). Lentiviral shRNA vectors targeting epidermal growth factor receptor (EGFR) and mitogen-activated protein kinase kinase

1 (MAP2K1), as well as a lentiviral overexpression vector for NFE2 like bZIP transcription factor 2 (NFE2L2), together with their corresponding control vectors, were supplied by Tsingke Biotechnology (China). Gene-specific primers for EGFR, MAP2K1, NFE2L2 and GAPDH were obtained from the PrimerBank (https://pga.mgh.harvard.edu/primerbank/index.html) and synthesized by Tsingke Biotechnology (China). Primary antibodies against EGFR (1:1000; Cat# A23381), MAP2K1 (1:1000; Cat# A19565), NFE2L2 (1:1000; Cat# A3577), GAPDH (1:50,000; Cat# A19056) and HRP-conjugated secondary antibody (1:5000; Cat# AS014) were provided by ABclonal (China).

Cell culture and chemical exposure

Human bladder cancer (BLCA) cell lines T24 and SW780 were both purchased from the American Type Culture Collection (ATCC, USA). T24 and SW780 cells were cultured under standard conditions. T24 cells were maintained in RPMI-1640 (Gibco, USA) supplemented with 10% fetal bovine serum (FBS; Hyclone, USA) and 1% penicillin-streptomycin (Invitrogen, USA). Cells were incubated at 37°C in a humidified atmosphere containing 5% CO_2. SW780 cells were propagated in Leibovitz's L-15 medium (Merck, USA) enriched with 10% FBS and 1% penicillin-streptomycin. The cells were incubated under identical temperature and CO_2 conditions as T24. For passaging, cells were dissociated with 0.25% trypsin-EDTA, monitored microscopically for detachment, and resuspended in fresh medium after

centrifugation. Both cell lines were routinely tested for mycoplasma contamination and maintained in logarithmic growth phase for experiments. Cell viability was confirmed via trypan blue exclusion (>95% viability) prior to experimental use.

PCP or 2,4-DCP was dissolved in DMSO and diluted to final concentrations of 0.05, 0.5 and 5 μM in serum-free medium. Cells were exposed to three tested concentrations for 24 hours, while control groups received an equivalent volume of DMSO (Wang et al. 2024). Post-treatment, cells were washed twice with PBS, harvested using 0.25% trypsin-EDTA, and processed for downstream analyses. For invasion and migration assays, the same drug concentrations were maintained in both upper and lower chambers. The final DMSO concentration was kept at 0.1% (v/v) in all PCP-treated and vehicle control groups.

Transfection assays

Short hairpin RNA (shRNA) constructs targeting EGFR and MAP2K1, as well as a lentiviral NFE2L2 overexpression vector, were designed and synthesized by Tsingke Biotechnology (China). The shRNAs were cloned into the pLKO.1 lentiviral backbone. The target sequences were 5'-AGAATGTGGAATACCTAAGG-3' (sense) and 5'-CCTTAGGTATTCCACATTCT-3' (antisense) for EGFR, and 5'-GCTTCTATGGTGCGTTCTACA-3' (sense) and 5'-TGTAGAACGCACCATAGAAGC-3' (antisense) for MAP2K1. For NFE2L2 gain-of-function, the full-length human NFE2L2 coding sequence (NM_006164) was

subcloned into a lentiviral expression vector, together with the corresponding empty vector as a control. Lentiviral particles were produced by co-transfecting HEK293T cells with individual transfer plasmids and the appropriate packaging and envelope plasmids using Lipofectamine 3000 (Thermo Fisher Scientific, USA) according to the manufacturer's instructions. Briefly, HEK293T cells were seeded in 6-well plates at a density of 2×10^5 cells/well 24 h prior to transfection to achieve 70–80% confluence at the time of transfection. For each well, 2.5 μg of transfer plasmid and 2.5 μg of packaging/envelope plasmid mix were diluted in 125 μL Opti-MEM reduced-serum medium, combined with 5 μL Lipofectamine 3000 and 5 μL P3000 reagent, and incubated at room temperature for 10 min before dropwise addition to cells. The final lipid-to-plasmid mass ratio was 2:1. Medium was replaced with fresh complete DMEM 6 h post-transfection. Viral supernatants were collected 48 h after transfection, clarified by centrifugation at 1000 × g for 5 min, and used to infect T24 and SW780 cells at a multiplicity of infection (MOI) of 10. Infected cells were subsequently selected with 2 μg/mL puromycin for 7 days to generate pooled stable EGFR- and MAP2K1-knockdown and NFE2L2-overexpressing cell populations, whereas cells infected with empty-vector virus served as negative controls. Knockdown and overexpression efficiencies were confirmed by real-time quantitative PCR (RT-qPCR).

Transwell invasion assay

Transwell inserts with polycarbonate membranes (8-μm pores; Corning, USA) were coated with Matrigel (1:8 dilution in serum-free medium; BD Biosciences, USA) and allowed to gel at 37 °C for 1 h. Cells ($5x10^4$ per well) in serum-free medium were seeded into the upper chambers, and 600 μL of medium containing 10% FBS was added to the lower chambers as a chemoattractant. After 24 h, non-invading cells on the upper membrane surface were gently removed with a cotton swab. Invaded cells on the underside were fixed with 4% paraformaldehyde, stained with 0.1% crystal violet, and quantified under an inverted microscope (Nikon, Japan) by counting five random fields per insert.

Wound healing assay

Cells were seeded in 6-well plates at $1x10^6$ cells/well and grown to a confluent monolayer (about 90% confluence). A linear scratch was created in the monolayer using a sterile 200-μL pipette tip. Detached cells were gently removed by washing with PBS, and cells were then cultured in serum-free medium. Wound closure was imaged at 0 h and 24 h, and migration distance was quantified using ImageJ.

Cell counting kit-8 (CCK-8) proliferation assay

Cells were plated at 3×10^3 cells/well in 96-well plates and incubated for 12–72 h.

At the indicated time points, 10 μL of CCK-8 reagent (Beyotime, China) was added to each well and incubated for 2 h. Absorbance at 450 nm was then measured using a microplate reader (BioTek, USA), and proliferation was expressed as absorbance values normalized to the untreated control group.

Western blot

Total protein was extracted using RIPA lysis buffer (Beyotime, China) containing protease inhibitors (Roche, USA). Protein concentrations were quantified via BCA assay (Beyotime, China). Equal amounts of protein were separated by 10% SDS-PAGE and transferred onto PVDF membranes (Millipore, USA). Membranes were blocked with 5% non-fat milk in TBST for 1 h at room temperature and incubated overnight at 4°C with primary antibodies against EGFR (1:1000; Cat# A23381, ABclonal), MAP2K1 (1:1000; Cat# A19565, ABclonal), NFE2L2 (1:1000; Cat# A3577, ABclonal). GAPDH (1:50000; Cat# A19056, ABclonal) served as the loading control. After washing, membranes were incubated with HRP-conjugated secondary antibodies (1:5000; Cat# AS014, ABclonal) for 1 h at room temperature. Protein bands were visualized using an ECL detection system (Beyotime, China) and quantified via ImageJ software.

RT-qPCR

Total RNA was extracted using TRIzol reagent (Thermo Fisher Scientific, USA) and treated with DNase I (Roche, Switzerland) to remove genomic DNA. RNA concentration and purity were assessed spectrophotometrically, and integrity was verified by agarose gel electrophoresis. Complementary DNA (cDNA) was synthesized from 1 μg RNA using the PrimeScript RT kit (Takara, Japan) according to the manufacturer's instructions. RT-qPCR was performed with TB Green Premix Ex Taq II (Takara) on an Applied Biosystems QuantStudio 1 real-time PCR system (Thermo Fisher Scientific) under standard two-step cycling (95 °C for 30 s; 40 cycles of 95 °C for 5 s and 60 °C for 30 s), followed by a melt-curve (65–95 °C) to confirm single products. Each 10-μL reaction contained 0.2 μM of each primer and approximately 1–2 ng cDNA. Primers were designed based on PrimerBank sequences and synthesized by Tsingke Biotechnology, and the sequences of all primers are provided as follows: EGFR, F: 5'- AGGCACGAGTAACAAGCTCAC-3', R: 5'-ATGAGGACATAACCAGCCACC-3'; MAP2K1, F: 5'-CAATGGCGGTGTGGTGTTC-3', R: 5'- GATTGCGGGTTTGATCTCCAG-3'; NFE2L2, F: 5'-TCAGCGACGGAAAGAGTATGA-3', R: 5'-CCACTGGTTTCTGACTGGATGT-3'; GAPDH, F: 5'-GGAGCGAGATCCCTCCAAAAT-3', R: 5'-GGCTGTTGTCATACTTCTCATGG-3'. Relative mRNA expression was calculated by the $2^{-\Delta\Delta Ct}$ method using GAPDH for normalization, with each condition run in triplicate across at least three independent experiments.

Actinomycin D (ActD) and cycloheximide (CHX) chase assays

To assess the effects of PCP on mRNA transcription and protein stability, ActD (Sigma-Aldrich, USA) and CHX (MedChemExpress, USA) chase assays were performed in SW780 cells.

For transcription inhibition experiments, SW780 cells were seeded in 6-well plates at a density of 2×10^5 cells/well and cultured overnight. Cells were pretreated with 5 μg/mL ActD for 1 h, followed by 9 h exposure to 0.5 μM PCP or DMSO. At the end of treatment, cells were harvested for RNA extraction. mRNA levels of EGFR, MAP2K1, and NFE2L2 were measured by RT-qPCR, with GAPDH as the internal control.

For protein stability assays, SW780 cells were treated with 0.5 μM PCP or DMSO for 24 h. Subsequently, 50 μg/mL CHX was added to block protein synthesis. Cells were harvested at 0, 6, and 12 h post-CHX addition. Protein levels of EGFR, MAP2K1, and NFE2L2 were measured by Western blot, with GAPDH as the loading control. Each experiment was performed with ≥ 3 biological replicates.

Immunohistochemistry analyses

The immunohistochemistry analyses of EGFR, MAP2K1 and NFE2L2 were obtained from the Human Protein Atlas (HPA, https://www.proteinatlas.org/).

Identification of PCP toxicity

The chemical structures of PCP were retrieved from the PubChem (https://pubchem.ncbi.nlm.nih.gov/). The *in-silico* carcinogenicity predictions were assessed through the ADMETLAB 3.0 (https://admetlab3.scbdd.com/), the ProTox3 (https://tox.charite.de/protox3/#) and ChemBlink (https://www.chemblink.com).

Transcriptome sequencing data collection and processing

The RNA sequencing (RNA-seq) data with count and FPKM formats of the BLCA samples from the Cancer Genome Atlas (TCGA) were downloaded from the UCSC Xena (https://xena.ucsc.edu/), where the follow-up duration and survival statuses were also downloaded. Another four BLCA cohorts, including GSE13507 (Kim et al. 2010), GSE31684 (Riester et al. 2012), GSE5287 (Als et al. 2007) and GSE32894 (Sjodahl et al. 2012), were downloaded from the Gene Expression Omnibus (GEO, https://ncbi.nlm.nih.gov/geo/), and the overall survival (OS) information of these BLCA cases were obtained from the GEO website or their published literature. The R software (v4.4.2) was used for data normalization and batch effect correction. The genes with the average expression level < 0.5 and the cases with the follow-up duration ≤ 30 days were removed. Subsequently, the RNA-seq data was transformed into log2(TPM + 1), and the transcriptome sequencing data from the microarrays was

transformed into log2(expression + 1) (Wu et al. 2022). To ensure comparability between TCGA RNA-sequencing data and four GEO microarray datasets (GSE13507, GSE31684, GSE5287, GSE32894), the transformed datasets were merged and harmonized using the ComBat algorithm (from the sva package in R) to adjust for non-biological technical variations (batch effects) arising from different platforms and studies.

Collection of the target genes of PCP

Potential targets of PCP were systematically identified using the TargetNet (http://targetnet.scbdd.com/), SwissTargetPrediction (http://www.swisstargetprediction.ch/), chEMBL (https://www.ebi.ac.uk/chembl/) and STICH (https://stitch.embl.de/). The gene data from these four databases were merged, duplicates were removed, and the final target gene sets for PCP were compiled.

Functional enrichment

Gene enrichment analysis was performed using Metascape (https://metascape.org). Terms with a nominal P value < 0.05 and Q value < 0.05 were considered statistically significant.

Collection of BLCA-associated genes

BLCA-related genes were collected from GeneCards (https://www.genecards.org/), OMIM (https://www.omim.org/), and the Therapeutic Target Database (TTD) (https://ttd.idrblab.cn/). In GeneCards, the terms “bladder cancer,” “bladder carcinoma,” and “malignant neoplasm of bladder” were queried, and genes with a relevance score ≥ the mean were retained. Using the same terms, OMIM and TTD were also queried. BLCA-related genes were identified from the TCGA-BLCA cohort. The analysis was conducted in two steps. First, we identified genes that were differentially expressed between tumor tissues and paracancerous tissues, applying a threshold of $|\log_2$fold change [FC]$| > 1$ and FDR < 0.05. Subsequently, from this set of differentially expressed genes, we further filtered for those significantly associated with OS through univariate Cox regression, using a statistical cutoff of FDR < 0.05. The final BLCA gene set combined database-derived and TCGA-derived genes.

Protein-protein interaction (PPI) network construction

The PPI network was constructed using the STRING database (https://cn.string-db.org/) with a minimum required combined score of 0.4 (Zhang 2022). The results were then visualized through the Cytoscape software (v3.8.0). The cytoHubba plugin in the Cytoscape software was used to measure the connectivity degree of the proteins in the PPI network.

Construction of the prognosis models using machine learning

To clarify the prognostic value of target genes associated with PCP, we employed the Mime R package to develop prognostic models utilizing its comprehensive framework integrating 101 machine learning algorithms. The selected genes were incorporated into the machine learning framework. This framework encompasses ten classical machine learning algorithms: Random Survival Forest (RSF), Elastic Net (Enet), Stepwise Cox (StepCox), CoxBoost, Partial Least Squares Regression for Cox (plsRcox), Supervised Principal Components (SuperPC), Generalized Boosted Regression Models (GBM), Survival Support Vector Machine (SurvivalSVM), Ridge, and Least Absolute Shrinkage and Selection Operator (Lasso). Variable selection was conducted using one of four embedded filters—Lasso, StepCox, CoxBoost, or RSF—each configured with specific core parameters. A total of 101 algorithm–filter combinations were systematically evaluated under a K-fold cross-validation scheme to construct models on the training dataset. The TCGA-BLCA cohort was selected as the training cohort, while the GSE13507, GSE31684, GSE5287, and GSE32894 cohorts were chosen for external validation. The final model was selected based on the highest mean concordance index (C-index) observed in the validation cohorts, representing the most robust predictive model with optimal accuracy and a reduced risk of overfitting. (Wu et al. 2025). The optimized model generated a PCP-related score (PCPRS) for each patient, defined as the model-derived linear predictor of

overall mortality risk. In the training cohort (TCGA-BLCA), the cohort median PCPRS was used as the cut-off to stratify all patients into low- and high-PCPRS groups. This prespecified cut-off was then applied unchanged to each external validation cohort.

Internal validation of the prognostic model

To evaluate the generalizability of PCPRS and correct for potential overfitting, two complementary resampling strategies were implemented in the TCGA-BLCA training cohort.

The caret R package was used to perform 10-fold cross-validation. Briefly, the dataset was randomly partitioned into 10 equal-sized subsets using the createFolds function to ensure reproducibility. In each iteration, 9 subsets were used for model training and the remaining subset was used for validation. This process was repeated 10 times until every sample had been used for validation exactly once. The apparent C-index was recorded for each fold, and the mean and standard deviation across 10 folds were reported as the cross-validated performance metric.

To further quantify and correct the optimism inherent in the apparent C-index, 1000 bootstrap resamples were generated using the rms R package. For each bootstrap sample, the same 101-algorithm machine learning screening pipeline was repeated to derive a bootstrap model. The performance of this bootstrap model was then

evaluated on both the bootstrap sample itself (apparent C-index) and the original TCGA-BLCA cohort (test C-index). The optimism for each resample was calculated as the difference between the apparent and test C-indices. After 1000 iterations, the average optimism was computed as the mean of all 1000 optimism values. The optimism-corrected C-index was obtained by subtracting the average optimism from the naive apparent C-index of the original model. This bootstrap-based internal validation procedure follows the methodology implemented in the validate function of the rms package and provides an unbiased estimate of model discrimination by accounting for overfitting.

Meta-analyses

Meta-analyses of hazard ratios (HRs) were performed using the meta package in R. HRs and their 95% confidence intervals (CIs) were derived from univariate Cox models fitted with the survival package. Between-study heterogeneity was assessed using Cochran's Q test. Substantial heterogeneity was defined as $P < 0.10$, in which case a random-effects model was applied, whereas a fixed-effect model was used when heterogeneity was not significant ($P \geq 0.10$) to pool the HRs. (Wang et al. 2012).

Detecting the molecular mechanisms associated with EGFR, MAP2K1 and NFE2L2 in BLCA

The Top 100 genes mostly correlated with EGFR, MAP2K1 and NFE2L2 were obtained from the Gene Expression Profiling Interactive Analysis (GEPIA, http://gepia.cancer-pku.cn/). Subsequently, gene functional enrichment was conducted, as mentioned above.

Molecular docking (MD)

MD was performed using PyMOL (v3.0.3), AutoDockTools (ADT) (v1.5.6), and AutoDock Vina (v1.2.5) to investigate the binding of PCP to EGFR (PDB 8F1X), MAP2K1 (PDB 3W8Q), and the NFE2L2(PDB 2FLU). Crystal structures were obtained from the RCSB PDB (https://www.rcsb.org/). Receptors were prepared in PyMOL/ADT by removing co-crystallized ligands and nonessential waters/heteroatoms while retaining catalytic or structural ions, adding polar hydrogens, and assigning Gasteiger partial charges; receptors were kept rigid and ligands treated as flexible. The PCP ligand was geometry-optimized with the MMFF94 force field in OpenBabel, converted to PDBQT in ADT, and rotatable bonds were defined.

The docking grid box was centered on the coordinates of the co-crystallized ligand (active site) and set to 28.0 × 28.0 × 28.0 $Å^3$ to fully encompass the binding pocket. Vina runs used an exhaustiveness of 120 and the default iterated global–local search

with BFGS local optimization. To assess robustness, each receptor–ligand pair was docked in ten independent runs with different random seeds; poses were ranked by predicted binding affinity (kcal/mol) from the Vina scoring function, and the lowest-energy pose was carried forward for analysis. Post-docking, poses were visually inspected in PyMOL and protein–ligand contacts annotated (hydrogen bonds, hydrophobic interactions). All structural figures were rendered in PyMOL.

Molecular dynamics simulations (MDS)

MDS were performed using GROMACS (v2022.4) to investigate the conformational dynamics and stability of the protein-ligand complex. Hydrogen atoms were added to the protein using the pdb2gmx tool with the AMBER99SB-ILDN force field, and protonation states of ionizable residues were adjusted to match physiological pH (7.4) using the PROPKA algorithm. The ligand topology was generated using the CHARMM General Force Field (CGenFF, v4.6) via the SwissParam server, and its parameters were validated through energy minimization with the steepest descent algorithm (convergence criterion: maximum force <1000 $kJ \cdot mol^{-1} \cdot nm^{-1}$, step size: 0.01 nm, maximum steps: 5000).

The solvated system was constructed by embedding the protein-ligand complex in a cubic simulation box filled with TIP3P water molecules, extending 1.0 nm from the solute surface in all directions. Counterions (Na^+/Cl^-) were added using the genion tool to neutralize the system to a net charge of 0 at an ionic concentration of 0.15 M,

mimicking physiological saline conditions. Energy minimization was performed using the steepest descent algorithm with the Verlet cutoff scheme (cutoff distance: 1.4 nm) to relax steric clashes, followed by two-phase equilibration: (1) NVT equilibration (constant particle number, volume, temperature): Performed for 100 ps with a 2-fs time step, using the velocity-rescaling thermostat (V-rescale) with a coupling constant of τ_T = 0.1 ps to maintain the system temperature at 310 K (37°C). (2) NPT equilibration (constant particle number, pressure, temperature): Performed for 200 ps with a 2-fs time step, using the Parrinello-Rahman barostat with a coupling constant of τ_P = 2.0 ps to maintain isotropic pressure at 1 atm. The compressibility was set to 4.5×10^{-5} bar^{-1}.

During equilibration, position restraints (force constant: 1000 $kJ\cdot mol^{-1}\cdot nm^{-2}$) were applied to the protein backbone and ligand heavy atoms to prevent large conformational deviations prior to production runs. Production MDS were performed for 100 ns with a 2-fs time step. Bond lengths involving hydrogen atoms were constrained using the LINCS algorithm (iterations: 4; expansion order: 2; tolerance: 1×10^{-4}) to enable stable integration. Long-range electrostatic interactions were calculated via the Particle Mesh Ewald (PME) method with a Fourier spacing of 0.12 nm and a Coulomb cutoff of 1.4 nm. Van der Waals interactions were truncated at 1.4 nm. Trajectory data were saved every 10 ps for subsequent analysis. Root mean square deviation (RMSD), root mean square fluctuation (RMSF), radius of gyration (Rg), solvent-accessible surface area (SASA), and hydrogen bond occupancy were

computed using GROMACS built-in tools (gmx rms, gmx rmsf, gmx gyrate, gmx sasa, gmx hbond, respectively).

Statistical analysis

All statistical analyses were performed using R software (v4.4.2) and GraphPad Prism (v8.0.1). Continuous variables are expressed as mean ± standard deviation (SD), and categorical variables as frequencies and percentages (n, %). Group comparisons for continuous variables were conducted using Welch's t-test or the Mann-Whitney U test, while associations between categorical variables were assessed by Chi-square or Fisher's exact test. Spearman correlation analyses were performed with the "cor.test" function in R. Principal component analysis (PCA) was performed using the "prcomp" function in R. Kaplan-Meier survival analysis with a log-rank test was conducted using the survival R package. A two-sided $P<0.05$ was considered statistically significant unless stated otherwise.

References

Als, A. B., L. Dyrskjot, H. von der Maase, K. Koed, F. Mansilla, H. E. Toldbod, J. L. Jensen, B. P. Ulhoi, L. Sengelov, K. M. Jensen, and T. F. Orntoft. 2007. "Emmprin and survivin predict response and survival following cisplatin-containing chemotherapy in patients with advanced bladder cancer." *Clin Cancer Res* 13 (15 Pt 1):4407-14. doi: 10.1158/1078-0432.CCR-07-0109.

Kim, W. J., E. J. Kim, S. K. Kim, Y. J. Kim, Y. S. Ha, P. Jeong, M. J. Kim, S. J. Yun, K. M. Lee, S. K. Moon, S. C. Lee, E. J. Cha, and S. C. Bae. 2010. "Predictive value of progression-related gene classifier in primary non-muscle invasive bladder cancer." *Mol Cancer* 9:3. doi: 10.1186/1476-4598-9-3.

Riester, M., J. M. Taylor, A. Feifer, T. Koppie, J. E. Rosenberg, R. J. Downey, B. H. Bochner, and F. Michor. 2012. "Combination of a novel gene expression signature with a clinical nomogram improves the prediction of survival in high-risk bladder cancer." *Clin Cancer Res* 18 (5):1323-33. doi: 10.1158/1078-0432.CCR-11-2271.

Sjodahl, G., M. Lauss, K. Lovgren, G. Chebil, S. Gudjonsson, S. Veerla, O. Patschan, M. Aine, M. Ferno, M. Ringner, W. Mansson, F. Liedberg, D. Lindgren, and M. Hoglund. 2012. "A molecular taxonomy for urothelial carcinoma." *Clin Cancer Res* 18 (12):3377-86. doi: 10.1158/1078-0432.CCR-12-0077-T.

Wang, L., L. Chen, D. Schlenk, F. Li, and J. Liu. 2024. "Parabens promotes invasive properties of multiple human cells: A potential cancer-associated adverse outcome pathway." *Sci Total Environ* 926:172015. doi: 10.1016/j.scitotenv.2024.172015.

Wang, X. T., W. Y. Wei, F. B. Kong, C. Lian, W. Luo, Q. Xiao, and Y. B. Xie. 2012. "Prognostic significance of Cdx2 immunohistochemical expression in gastric cancer: a meta-analysis of published literatures." *J Exp Clin Cancer Res* 31 (1):98. doi: 10.1186/1756-9966-31-98.

Wu, J., Q. Bi, X. Zheng, H. Cao, C. Hao, Z. Sun, and W. Wang. 2022. "STEAP3 can

predict the prognosis and shape the tumor microenvironment of clear cell renal cell carcinoma." *BMC Cancer* 22 (1):1204. doi: 10.1186/s12885-022-10313-z.

Wu, Y., Y. Cui, X. Zheng, X. Yao, and G. Sun. 2025. "Integrated machine learning to predict the prognosis of lung adenocarcinoma patients based on SARS-COV-2 and lung adenocarcinoma crosstalk genes." *Cancer Sci* 116 (1):95-111. doi: 10.1111/cas.16384.

Zhang, Z. F. 2022. "A comprehensive prognostic and immune infiltration analysis of EXOC3L1 in pan-cancer." *Front Genet* 13:1044100. doi: 10.3389/fgene.2022.1044100.

3. The R code used in this study

```
#Reduce the batch effects using the sva package

library(limma)

library(sva)

files=dir()

files=grep("normalize.txt$", files, value=T)

geneList=list()

for(file in files){
```

```
        if(file=="merge.preNorm.txt"){next}
        if(file=="merge.normalize.txt"){next}
        rt=read.table(file, header=T, sep="\t", check.names=F)
        geneNames=as.vector(rt[,1])
        uniqGene=unique(geneNames)
        header=unlist(strsplit(file, "\\.|\\-"))
        geneList[[header[1]]]=uniqGene
}
interGenes=Reduce(intersect, geneList)
allTab=data.frame()
batchType=c()
for(i in 1:length(files)){
        inputFile=files[i]
        if(inputFile=="merge.preNorm.txt"){next}
        if(inputFile=="merge.normalize.txt"){next}
        header=unlist(strsplit(inputFile, "\\.|\\-"))
        rt=read.table(inputFile, header=T, sep="\t", check.names=F)
```

```
        rt=as.matrix(rt)
        rownames(rt)=rt[,1]
        exp=rt[,2:ncol(rt)]
        dimnames=list(rownames(exp),colnames(exp))
        data=matrix(as.numeric(as.matrix(exp)),nrow=nrow(exp),dimnames=dimnames)
        rt=avereps(data)
        colnames(rt)=paste0(header[1], "_", colnames(rt))
        if(i==1){
         allTab=rt[interGenes,]
        }else{
         allTab=cbind(allTab, rt[interGenes,])
        }
        batchType=c(batchType, rep(i,ncol(rt)))
}
outTab=rbind(geneNames=colnames(allTab), allTab)
write.table(outTab, file="merge.preNorm.txt", sep="\t", quote=F, col.names=F)
outTab=ComBat(allTab, batchType, par.prior=TRUE)
```

```
outTab=rbind(geneNames=colnames(outTab), outTab)

write.table(outTab, file="merge.normalize.txt", sep="\t", quote=F, col.names=F)
```

#Prognosis model construction.

```
library(Mime1)

library(survival)

library(dplyr)

tcga <- read.table(

  "INPUT_TCGA.txt",

  header = TRUE,

  sep = "\t",

  stringsAsFactors = FALSE,

  check.names = FALSE

)

colnames(tcga)[match(c("futime", "fustat"), colnames(tcga))] <- c("OS.time", "OS")

tcga$SAMPLE_ID <- tcga$sample

train_data <- list(
```

```
    train = tcga[, c("OS.time", "OS", setdiff(colnames(tcga), c("sample", "OS.time", "OS")))]
)
geo <- read.table(
    "INPUT_GEO.txt",
    header = TRUE,
    sep = "\t",
    stringsAsFactors = FALSE,
    check.names = FALSE
)
colnames(geo)[match(c("futime", "fustat"), colnames(geo))] <- c("OS.time", "OS")
common_genes <- intersect(
    colnames(train_data$train),
    colnames(geo)
)
geo <- geo[, common_genes]
test_data <- list(
    test = geo
```

```
)
uni_cox <- function(expr, time, status) {
  pvals <- apply(expr, 2, function(x) {
    cox <- coxph(Surv(time, status) ~ x)
    summary(cox)$coefficients[,"Pr(>|z|)"]
  })
  names(pvals)
}
sig_genes <- uni_cox(
  expr = train_data$train[, !(colnames(train_data$train) %in% c("OS.time", "OS"))],
  time = train_data$train$OS.time,
  status = train_data$train$OS
)
sig_genes <- sig_genes[which(sig_genes < 1)]
train_data$train <- train_data$train[, c("OS.time", "OS", sig_genes)]
test_data$test    <- test_data$test[, c("OS.time", "OS", sig_genes)]
ml_res <- ML.Dev.Prog.Sig(
```

```
    train = train_data$train,
    test    = test_data$test,
    mode    = "all",
    pre_filt = TRUE,
    nfolds = 10,
    cores = 4
)
cindex_dis_all(ml_res)
best_model_name <- names(which.max(ml_res$cindex_test))
best_model <- ml_res$model[[best_model_name]]
saveRDS(ml_res, file = "Mime1_101ML_Prognostic_Model.rds")
write.table(
    ml_res$risk_score_test[[best_model_name]],
    file = "GEO_RiskScore.txt",
    quote = FALSE,
    sep = "\t"
)
```

# C-index calculation

```
library(survival)
library(dplyr)
library(tidyr)
library(rms)
dat <- read.table("RISK_ALL.txt", header = TRUE, sep = "\t",
                  stringsAsFactors = FALSE)
head(dat)
summary_table <- dat %>%
  group_by(Cohort) %>%
  summarise(
    N = n(),
    Mean_PCPRS = mean(PCPRS, na.rm = TRUE),
    SD_PCPRS    = sd(PCPRS, na.rm = TRUE),
    Low_n        = sum(risk == "low", na.rm = TRUE),
    Low_pct      = Low_n / N * 100,
```

```
    High_n         = sum(risk == "high", na.rm = TRUE),
    High_pct      = High_n / N * 100
  )
print(summary_table)
calc_cindex <- function(data) {
  survConcordance(Surv(futime, fustat) ~ PCPRS, data = data)$concordance
}
B <- 200
boot_results <- dat %>%
  group_by(Cohort) %>%
  summarise(
    C_index = calc_cindex(cur_data()),
    boot_ci = list({
      c_vec <- replicate(B, {
        idx <- sample(1:nrow(cur_data()), replace = TRUE)
        boot_dat <- cur_data()[idx, ]
        calc_cindex(boot_dat)
```

```
      })
      quantile(c_vec, probs = c(0.025, 0.975), na.rm = TRUE)
    })
  ) %>%
  mutate(
    lower_95 = sapply(boot_ci, function(x) x[1]),
    upper_95 = sapply(boot_ci, function(x) x[2])
  ) %>%
  select(-boot_ci)
print(boot_results)
tcga <- subset(dat, Cohort == "TCGA-BLCA")
n <- nrow(tcga)
folds <- sample(rep(1:10, length.out = n))
cv_c <- numeric(10)
for (k in 1:10) {
  train <- tcga[folds != k, ]
  test   <- tcga[folds == k, ]
```

```
    fit <- coxph(Surv(futime, fustat) ~ PCPRS, data = train)

    lp <- predict(fit, newdata = test, type = "lp")

    cv_c[k] <- survConcordance(Surv(futime, fustat) ~ lp, data = test)$concordance

}

cv_mean <- mean(cv_c)

cv_sd      <- sd(cv_c)
```

#Calibration plot

```
library(survival)

library(riskRegression)

library(ggplot2)

library(dplyr)

library(tidyr)

data <- read.table("RISK_ALL.txt", header = TRUE, sep = "\t", stringsAsFactors = FALSE)

time_point <- 1095

#time_point <- 365

get_calibration_data <- function(df, tp) {
```

```
cox_fit <- coxph(Surv(futime, fustat) ~ PCPRS, data = df, x = TRUE)

pred <- predictCox(cox_fit, newdata = df, times = tp, type = "survival")

df$pred_prob <- pred$survival[, 1]

n_obs <- nrow(df)

n_groups <- min(10, max(3, floor(n_obs / 10)))

breaks <- quantile(df$pred_prob, probs = seq(0, 1, length.out = n_groups + 1),

                   na.rm = TRUE)

if (length(unique(breaks)) < 2) {

  breaks <- unique(breaks)

} else {

  breaks <- unique(breaks)

}

if (length(breaks) < 2) {

  return(data.frame())

}

df$group <- cut(df$pred_prob, breaks = breaks, include.lowest = TRUE)

group_stats <- df %>%
```

```
    group_by(group) %>%
    summarise(mean_pred = mean(pred_prob, na.rm = TRUE),
              n = n()) %>%
    ungroup()
  obs_prob <- sapply(split(df, df$group), function(g) {
    if (nrow(g) < 3) return(NA)
    km_fit <- survfit(Surv(futime, fustat) ~ 1, data = g)
    sum_km <- summary(km_fit, times = tp, extend = TRUE)
    if (length(sum_km$surv) > 0) sum_km$surv[1] else NA
  })
  group_stats$obs_prob <- obs_prob[group_stats$group]
  group_stats <- group_stats[!is.na(group_stats$obs_prob), ]
  return(group_stats)
}
cohorts <- unique(data$Cohort)
plot_list <- list()
for (coh in cohorts) {
```

```
df_sub <- subset(data, Cohort == coh)

cat("Processing cohort:", coh, "    n=", nrow(df_sub), "\n")

if (nrow(df_sub) < 10) {

    warning(paste("Skipping", coh, "due to small sample size (<10)"))

    next

}

cal_data <- get_calibration_data(df_sub, time_point)

if (nrow(cal_data) < 2) {

    warning(paste("Insufficient groups for", coh))

    next

}

p <- ggplot(cal_data, aes(x = mean_pred, y = obs_prob)) +

    geom_point(size = 3, color = "blue") +

    geom_abline(intercept = 0, slope = 1, linetype = "dashed", color = "red", size = 1) +

    xlim(0, 1) + ylim(0, 1) +

    labs(

        title = paste("Calibration Plot -", coh),
```

```
        subtitle = paste("At", time_point, "days (", round(time_point/365.25, 1), "years)"),
        x = "Mean Predicted Survival Probability",
        y = "Observed Survival Probability (KM)"
      ) +
      theme_bw(base_size = 12) +
      theme(plot.title = element_text(hjust = 0.5),
            plot.subtitle = element_text(hjust = 0.5))
    plot_list[[coh]] <- p
}
if (length(plot_list) > 0) {
    pdf("Calibration_plots_by_cohort.pdf", width = 7, height = 6)
    for (p in plot_list) {
      print(p)
    }
    dev.off()
    cat("PDF saved as 'Calibration_plots.pdf'\n")
} else {
```

```
    cat("No plots generated. Check data or time point.\n")
}


#post hoc power analysis
library(survival)
data <- read.table("risk_GSE5287.txt", header = TRUE, row.names = 1)
head(data)
data$risk <- factor(data$risk, levels = c("low", "high"))
cox_fit <- coxph(Surv(futime, fustat) ~ risk, data = data)
summary(cox_fit)
hr <- exp(coef(cox_fit))[1]
log_hr <- coef(cox_fit)[1]
se_log_hr <- sqrt(diag(vcov(cox_fit)))[1]
z_stat <- log_hr / se_log_hr
alpha <- 0.05
z_alpha <- qnorm(1 - alpha/2)
power <- 1 - pnorm(z_alpha - abs(z_stat)) + pnorm(-z_alpha - abs(z_stat))
```

```
cat("Z：", round(z_stat, 3), "\n")

cat("post hoc power（α = 0.05）：", round(power, 4), "\n")
```

#meta analysis

```
library(meta)

dat <- read.table("/data/inputs/input_C.txt",

                  header = TRUE,

                  sep = "\t")

str(dat)

dat$logHR <- log(dat$HR)

dat$logHR.95L <- log(dat$HR.95L)

dat$logHR.95H <- log(dat$HR.95H)

dat$se <- (dat$logHR.95H - dat$logHR.95L) / (2 * 1.96)

meta_fixed <- metagen(

  TE = logHR,

  seTE = se,

  data = dat,
```

```
  studlab = Cohort,
  sm = "HR",
  fixed = TRUE,
  random = FALSE
)
meta_fixed
meta_fixed$Q
meta_fixed$I2
meta_fem <- metagen(
  TE = logHR,
  seTE = se,
  data = dat,
  studlab = Cohort,
  sm = "HR",
  fixed = TRUE,
  random = FALSE
)
```

```
summary(meta_fem)

meta_rem <- metagen(

  TE = logHR,

  seTE = se,

  data = dat,

  studlab = Cohort,

  sm = "HR",

  fixed = FALSE,

  random = TRUE,

  method.tau = "DL"     # DerSimonian-Laird

)

summary(meta_rem)

forest(meta_rem,

       sortvar = TE,

       xlab = "Hazard Ratio (HR)")
```

4. Supplementary Tables

Table S5 Importance of the BLCA-PCP genes in the PPI network.

Gene Symbol	MCC	DMNC	MNC	Degree	EPC	BottleNeck	EcCentricity	Closeness	Radiality	Betweenness	Stress	ClusteringCoefficient
TP53	8.50E+11	0.68694	45	45	19.439	8	0.5	50	3.90909	243.5933	1550	0.44848
CASP3	8.50E+11	0.72372	42	42	19.116	1	0.5	48.5	3.85455	163.36376	1242	0.48316
HSP90AA1	8.50E+11	0.71773	41	41	19.084	3	0.33333	47.83333	3.81818	166.34968	1114	0.48293
ESR1	8.50E+11	0.74094	40	40	18.944	12	0.5	47.5	3.81818	172.78303	1196	0.50256
PPARG	4.30E+11	0.70533	38	38	18.812	2	0.33333	46.33333	3.76364	167.05335	1102	0.48649
EGFR	8.49E+11	0.76308	38	38	18.509	1	0.5	46.5	3.78182	139.041	1014	0.52632
NFKB1	8.50E+11	0.73421	38	38	17.891	1	0.33333	46.33333	3.76364	150.50521	924	0.5064
PTGS2	7.21E+11	0.72123	36	36	18.204	7	0.33333	45.33333	3.72727	131.00379	1040	0.50635
MMP9	7.86E+11	0.80729	34	34	18.172	3	0.5	44.5	3.70909	75.19415	636	0.57754
ERBB2	4.39E+11	0.81759	32	32	17.261	1	0.5	43.5	3.67273	109.74654	764	0.59677
PARP1	8.46E+11	0.92165	30	30	17.205	1	0.33333	42.33333	3.61818	28.87247	370	0.68736
MDM2	4.61E+11	0.91755	29	29	17.176	1	0.33333	41.83333	3.6	28.29571	308	0.69212
PIK3CA	6.38E+10	0.88121	27	27	16.405	2	0.33333	40.83333	3.56364	24.29605	276	0.68091
MAPK1	3.94E+11	0.88456	26	26	16.504	1	0.33333	40.33333	3.54545	24.79669	254	0.69231
NFE2L2	7.85E+11	0.90773	25	25	15.938	2	0.33333	39.83333	3.52727	19.62283	204	0.72
CASP9	7.84E+11	0.98757	25	25	15.962	1	0.33333	39.83333	3.52727	11.90806	164	0.78333
MAPK14	3.89E+11	0.89638	24	24	15.766	2	0.33333	39.33333	3.50909	52.71734	368	0.72101

MCL1	4.07E+11	0.96395	24	24	15.606	1	0.33333	39.33333	3.50909	12.45532	168	0.77536
MAP2K1	7.85E+11	0.98785	23	23	15.527	1	0.33333	38.83333	3.49091	9.57053	120	0.80632
CDK1	3.90E+10	0.9505	22	22	14.94	1	0.33333	38	3.43636	9.59826	116	0.78788
CDK2	1.65E+10	0.95572	22	22	14.449	1	0.33333	38.33333	3.47273	9.43626	148	0.79221
ESR2	3.60E+09	0.85127	22	22	14.834	1	0.33333	38.33333	3.47273	20.99028	202	0.70563
AR	5.28E+10	0.95572	22	22	15.356	1	0.33333	38.33333	3.47273	14.93281	142	0.79221
CYP19A1	2220912	0.63868	20	20	13.175	2	0.33333	37.33333	3.43636	45.55535	424	0.54737
TERT	5.08E+10	1.00101	20	20	14.7	1	0.33333	37.16667	3.41818	4.25237	62	0.85789
CYP1A1	144600	0.6571	20	20	12.562	1	0.33333	37.16667	3.41818	41.90641	388	0.56316
ABCB1	6.27E+09	0.76764	20	20	13.341	1	0.33333	37.33333	3.43636	29.28857	332	0.65789
ABCG2	4.36E+07	0.72723	18	18	12.645	1	0.33333	36.33333	3.4	23.87913	308	0.64706
FLT1	8111640	0.80144	17	17	12.556	1	0.5	36	3.4	12.92092	122	0.72794
DNMT1	2.54E+10	0.95525	17	17	12.917	1	0.33333	35.83333	3.38182	4.41781	44	0.86765
CHEK2	2.61E+08	0.96921	16	16	12.782	1	0.33333	34.83333	3.30909	1.88273	28	0.9
NOS3	7706160	0.82122	15	15	11.668	1	0.33333	34.66667	3.32727	4.7161	48	0.78095
PDGFRA	1.49E+07	0.80119	15	15	11.981	1	0.33333	34.83333	3.34545	13.20783	76	0.7619
PTGS1	85992	0.59088	15	15	11.239	1	0.33333	34.83333	3.34545	23.31889	240	0.5619
CYP2E1	5448	0.65097	15	15	10.485	1	0.33333	34.66667	3.32727	12.75987	138	0.61905
CYP27B1	2640	0.51801	14	14	10.331	2	0.33333	34.16667	3.30909	23.74954	176	0.50549
CYP2C9	5760	0.64188	14	14	9.053	1	0.33333	34	3.29091	12.17873	128	0.62637
PIK3CB	164280	0.77916	13	13	11.139	1	0.33333	33.5	3.27273	3.15974	42	0.78205

PLAU	4.36E+07	0.90689	13	13	12.094	1	0.33333	33.83333	3.30909	1.33011	14	0.91026
CYP1A2	5952	0.67697	13	13	9.276	1	0.33333	33.5	3.27273	11.57213	120	0.67949
NOS2	7620480	0.90737	12	12	10.632	1	0.33333	33.16667	3.27273	0.71566	10	0.93939
CYP2C19	3240	0.6293	12	12	7.98	1	0.25	31.41667	3.07273	7.96251	54	0.65152
LCK	404640	0.83144	11	11	10.064	1	0.33333	32.33333	3.21818	1.12901	20	0.89091
PPARD	403320	0.7975	11	11	10.043	1	0.33333	32.66667	3.25455	1.89928	16	0.85455
PLK1	7257600	0.91628	11	11	10.062	1	0.33333	32	3.18182	0.10526	2	0.98182
LMNA	403200	0.85796	10	10	9.302	1	0.33333	31.83333	3.2	0.25146	6	0.95556
ALOX5	288	0.54893	9	9	6.911	1	0.33333	31.33333	3.18182	5.79387	70	0.63889
CYP2D6	1584	0.69213	9	9	6.121	1	0.33333	30.66667	3.10909	1.67711	28	0.80556
CA9	5041	0.76834	7	8	7.828	2	0.33333	31.16667	3.2	108	548	0.75
HPGD	48	0.52304	6	6	5.601	1	0.25	28.91667	3.01818	1.18519	10	0.73333
AHR	72	0.57059	6	6	5.635	1	0.33333	29.5	3.09091	0.91515	6	0.8
SDHB	4	0.30898	3	3	3.668	1	0.33333	27.83333	3.01818	0.14286	2	0.66667
TARDBP	6	0.46346	3	3	4.113	1	0.33333	28	3.03636	0	0	1
HTR2B	2	0.30779	2	2	3.091	1	0.33333	25.33333	2.78182	0	0	1
ADORA1	2	0.30779	2	2	3.231	1	0.33333	26.66667	2.92727	0	0	1
CA14	1	0	1	1	1.42	1	0.25	20	2.21818	0	0	0

Table S6 The baseline clinicopathological features of the BLCA cohorts.

Parameters	TCGA-BLCA (N=399)	GSE5287 (N=27)	GSE13507 (N=165)	GSE31684 (N=90)	GSE32894 (N=221)
Survival status					
Alive	223 (55.89%)	5 (18.52%)	96 (58.18%)	27 (30.00%)	196 (88.69%)
Dead	176 (44.11%)	22 (81.48%)	69 (41.82%)	63 (70.00%)	25 (11.31%)
Follow-up (day)	825.53 ± 835.05	114.44 ± 114.29	1451.45 ± 1131.13	1471.24 ± 1339.92	1213.05 ± 759.96
Age (year)	68.01 ± 10.66	-	65.18 ± 11.97	69.00 ± 10.18	69.41 ± 11.25
Sex					
Female	106 (26.57%)	-	30 (18.18%)	24 (26.67%)	60 (27.15%)
Male	293 (73.43%)	-	135 (81.82%)	66 (73.33%)	161 (72.85%)
TNM Stage					
0a	0 (0.00%)	-	23 (13.94%)	-	-
I	2 (0.50%)	-	80 (48.48%)	-	-
II	125 (31.33%)	-	26 (15.76%)	-	-
III	138 (34.59%)	-	21 (12.73%)	-	-
IV	132 (33.08%)	-	15 (9.09%)	-	-
Unknown	2 (0.50%)	-	0 (0.00%)	-	-
pT stage					
Ta	0 (0.00%)	-	24 (14.55%)	5 (5.56%)	109 (49.32%)
T1	4 (1.00%)	-	80 (48.48%)	10 (11.11%)	61 (27.60%)
T2	114 (28.57%)	-	31 (18.79%)	17 (18.89%)	43 (19.46%)
T3	191 (47.87%)	-	19 (11.52%)	40 (44.44%)	7 (3.17%)
T4	58 (14.54%)	-	11 (6.67%)	18 (20.00%)	1 (0.45%)
Unknown	32 (8.02%)	-	0 (0.00%)	0 (0.00%)	0 (0.00%)
M stage					
M0	189 (47.37%)	-	158 (95.76%)	56 (62.22%)	-
M1	10 (2.51%)	-	7 (4.24%)	34 (37.78%)	-
Unknown	200 (50.13%)	-	0 (0.00%)	0 (0.00%)	-
pN stage					
N0	230 (57.64%)	-	149 (90.3%)	-	27 (12.22%)
N1	46 (11.53%)	-	8 (4.85%)	-	3 (1.36%)
N2	75 (18.80%)	-	6 (3.64%)	-	10 (4.52%)
N3	7 (1.75%)	-	1 (0.61%)	-	0 (0.00%)
Unknown	41 (10.28%)	-	1 (0.61%)	-	181 (81.9%)
Grade					
High	378 (94.74%)	-	60 (36.36%)	84 (93.33%)	-
Low	18 (4.51%)	-	105 (63.64%)	6 (6.67%)	-
Unknown	3 (0.75%)	-	0 (0.00%)	0 (0.00%)	-

Table S7 The C-indexes of the 101 algorithms in the BLCA cohorts.

Method	TCGA-BLCA	GSE5287	GSE13507	GSE31684	GSE32894
CoxBoost	0.664	0.598	0.51	0.515	0.568
CoxBoost+Enet[alpha=0.1]	0.664	0.592	0.505	0.497	0.548
CoxBoost+Enet[alpha=0.2]	0.663	0.592	0.505	0.498	0.553
CoxBoost+Enet[alpha=0.3]	0.663	0.592	0.506	0.498	0.554
CoxBoost+Enet[alpha=0.4]	0.663	0.592	0.506	0.498	0.549
CoxBoost+Enet[alpha=0.5]	0.663	0.595	0.506	0.498	0.552
CoxBoost+Enet[alpha=0.6]	0.664	0.595	0.504	0.5	0.545
CoxBoost+Enet[alpha=0.7]	0.664	0.595	0.504	0.501	0.545
CoxBoost+Enet[alpha=0.8]	0.664	0.595	0.504	0.5	0.546
CoxBoost+Enet[alpha=0.9]	0.664	0.595	0.505	0.501	0.548
CoxBoost+GBM	0.559	0.537	0.515	0.516	0.647
CoxBoost+Lasso	0.664	0.595	0.504	0.501	0.546
CoxBoost+plsRcox	0.651	0.609	0.507	0.506	0.532
CoxBoost+Ridge	0.663	0.417	0.507	0.5	0.552
CoxBoost+RSF	0.574	0.527	0.511	0.525	0.606
CoxBoost+StepCox[backward]	0.653	0.618	0.488	0.517	0.535
CoxBoost+StepCox[both]	0.653	0.618	0.488	0.517	0.535
CoxBoost+StepCox[forward]	0.662	0.598	0.503	0.499	0.534
CoxBoost+SuperPC	0.609	0.521	0.601	0.513	0.768
CoxBoost+survivalSVM	0.531	0.583	0.548	0.51	0.526
Enet[alpha=0.1]	0.66	0.426	0.51	0.508	0.58
Enet[alpha=0.2]	0.657	0.414	0.509	0.508	0.582
Enet[alpha=0.3]	0.658	0.589	0.51	0.507	0.576
Enet[alpha=0.4]	0.657	0.589	0.51	0.508	0.581
Enet[alpha=0.5]	0.657	0.589	0.51	0.509	0.578
Enet[alpha=0.6]	0.66	0.598	0.51	0.505	0.57
Enet[alpha=0.7]	0.658	0.592	0.51	0.504	0.574
Enet[alpha=0.8]	0.662	0.598	0.511	0.505	0.569
Enet[alpha=0.9]	0.662	0.598	0.51	0.505	0.569
GBM	0.535	0.607	0.518	0.486	0.675
Lasso	0.662	0.595	0.51	0.504	0.569
Lasso+CoxBoost	0.664	0.592	0.502	0.507	0.55
Lasso+GBM	0.552	0.537	0.516	0.517	0.65
Lasso+plsRcox	0.651	0.609	0.507	0.506	0.532
Lasso+RSF	0.535	0.559	0.494	0.526	0.703
Lasso+StepCox[backward]	0.653	0.618	0.488	0.517	0.535
Lasso+StepCox[both]	0.653	0.618	0.488	0.517	0.535
Lasso+StepCox[forward]	0.662	0.598	0.503	0.499	0.534
Lasso+SuperPC	0.609	0.521	0.601	0.513	0.768

Lasso+survivalSVM	0.531	0.583	0.548	0.51	0.526
plsRcox	0.653	0.58	0.507	0.511	0.538
Ridge	0.672	0.604	0.506	0.519	0.6
RSF	0.527	0.513	0.464	0.524	0.501
RSF+CoxBoost	0.624	0.589	0.614	0.563	0.678
RSF+Enet[alpha=0.1]	0.624	0.586	0.614	0.562	0.678
RSF+Enet[alpha=0.2]	0.624	0.586	0.614	0.562	0.678
RSF+Enet[alpha=0.3]	0.624	0.586	0.614	0.562	0.679
RSF+Enet[alpha=0.4]	0.625	0.583	0.614	0.561	0.678
RSF+Enet[alpha=0.5]	0.625	0.583	0.614	0.561	0.678
RSF+Enet[alpha=0.6]	0.624	0.586	0.614	0.562	0.679
RSF+Enet[alpha=0.7]	0.625	0.583	0.614	0.561	0.678
RSF+Enet[alpha=0.8]	0.624	0.586	0.614	0.562	0.68
RSF+Enet[alpha=0.9]	0.625	0.583	0.614	0.561	0.678
RSF+GBM	0.512	0.509	0.515	0.499	0.5
RSF+Lasso	0.625	0.583	0.614	0.562	0.678
RSF+plsRcox	0.624	0.589	0.614	0.558	0.678
RSF+Ridge	0.623	0.583	0.614	0.564	0.678
RSF+StepCox[backward]	0.626	0.589	0.614	0.562	0.678
RSF+StepCox[both]	0.628	0.591	0.617	0.561	0.677
RSF+StepCox[forward]	0.625	0.589	0.615	0.561	0.679
RSF+SuperPC	0.531	0.583	0.548	0.51	0.526
RSF+survivalSVM	0.576	0.559	0.577	0.545	0.596
StepCox[backward]	0.645	0.58	0.512	0.528	0.518
StepCox[backward]+CoxBoost	0.647	0.583	0.51	0.529	0.522
StepCox[backward]+Enet[alpha=0.1]	0.652	0.583	0.509	0.533	0.528
StepCox[backward]+Enet[alpha=0.2]	0.65	0.586	0.509	0.53	0.527
StepCox[backward]+Enet[alpha=0.3]	0.65	0.586	0.509	0.531	0.527
StepCox[backward]+Enet[alpha=0.4]	0.649	0.586	0.509	0.53	0.526
StepCox[backward]+Enet[alpha=0.5]	0.65	0.586	0.509	0.531	0.527
StepCox[backward]+Enet[alpha=0.6]	0.647	0.586	0.51	0.531	0.524
StepCox[backward]+Enet[alpha=0.7]	0.649	0.586	0.51	0.531	0.526
StepCox[backward]+Enet[alpha=0.8]	0.647	0.586	0.511	0.531	0.524
StepCox[backward]+Enet[alpha=0.9]	0.648	0.586	0.51	0.53	0.524

StepCox[backward]+GBM	0.506	0.538	0.507	0.521	0.569
StepCox[backward]+Lasso	0.647	0.586	0.51	0.529	0.524
StepCox[backward]+plsRcox	0.651	0.609	0.513	0.529	0.537
StepCox[backward]+Ridge	0.653	0.423	0.508	0.535	0.53
StepCox[backward]+RSF	0.513	0.518	0.501	0.704	0.512
StepCox[backward]+SuperPC	0.596	0.547	0.598	0.514	0.762
StepCox[backward]+survivalSVM	0.538	0.494	0.527	0.489	0.596
StepCox[both]	0.645	0.58	0.512	0.528	0.518
StepCox[both]+CoxBoost	0.647	0.583	0.51	0.528	0.522
StepCox[both]+Enet[alpha=0.1]	0.649	0.586	0.509	0.53	0.524
StepCox[both]+Enet[alpha=0.2]	0.65	0.586	0.509	0.53	0.527
StepCox[both]+Enet[alpha=0.3]	0.649	0.586	0.509	0.531	0.526
StepCox[both]+Enet[alpha=0.4]	0.653	0.583	0.492	0.534	0.53
StepCox[both]+Enet[alpha=0.5]	0.649	0.586	0.51	0.531	0.526
StepCox[both]+Enet[alpha=0.6]	0.648	0.586	0.51	0.531	0.524
StepCox[both]+Enet[alpha=0.7]	0.648	0.586	0.51	0.531	0.524
StepCox[both]+Enet[alpha=0.8]	0.646	0.583	0.511	0.529	0.519
StepCox[both]+Enet[alpha=0.9]	0.649	0.586	0.51	0.531	0.526
StepCox[both]+GBM	0.519	0.506	0.506	0.521	0.536
StepCox[both]+Lasso	0.648	0.586	0.51	0.53	0.524
StepCox[both]+plsRcox	0.651	0.609	0.513	0.529	0.537
StepCox[both]+Ridge	0.654	0.426	0.508	0.536	0.533
StepCox[both]+RSF	0.501	0.547	0.463	0.526	0.602
StepCox[both]+SuperPC	0.596	0.547	0.598	0.514	0.762
StepCox[both]+survivalSVM	0.538	0.494	0.527	0.489	0.596
StepCox[forward]	0.674	0.589	0.494	0.561	0.535
SuperPC	0.608	0.518	0.603	0.513	0.774
survivalSVM	0.517	0.571	0.553	0.502	0.667
